# Identifying pyroptosis-hub genes and immune infiltration in neonatal hypoxic-ischemic brain injury

**DOI:** 10.3389/fimmu.2025.1616312

**Published:** 2025-09-05

**Authors:** Chi Qin, Yue Li, Meiying Cheng, Huixian Li, Ronghao Mu, Jian Jin, Bohao Zhang, Xin Zhao, Xiaoan Zhang

**Affiliations:** ^1^ Department of Radiology, The Third Affiliated Hospital of Zhengzhou University, Zhengzhou, China; ^2^ Department of Clinical Research and Translational Medicine, The Third Affiliated Hospital of Zhengzhou University, Zhengzhou, China; ^3^ Department of Child Developmental Behavior, The Third Affiliated Hospital of Zhengzhou University, Zhengzhou, China

**Keywords:** hypoxic-ischemic brain damage, pyroptosis, immune infiltration, bioinformatics, machine learning

## Abstract

**Background:**

Hypoxic-ischemic encephalopathy (HIE) is a leading cause of neonatal brain injury and neurodevelopmental disorders. Pyroptosis, an inflammatory programmed cell death, may offer new therapeutic targets for HIE by modulating cytokine expression and related pathways. This study aims to identify HIE-associated pyroptosis genes and explore potential drugs and molecular mechanisms.

**Methods:**

The gene microarray data of hypoxic-ischemic brain damage (HIBD) were obtained from the Gene Expression Omnibus (GEO) database. The Limma package was used to identify differentially expressed genes (DEGs). Weighted gene co-expression network analysis (WGCNA) was performed to find significant expression modules. GO and KEGG analyses were carried out for the pathway enrichment of DEGs, as well as protein–protein interaction (PPI) network analysis were subsequently conducted. Cytohubba software was employed to identify hub genes among DEGs. A random forest (RF) model assessed the pyroptosis-related genes, examining their diagnostic performance. Potential therapeutic drugs or compounds targeting the hub genes were screened through DSigDB, and their binding scores and affinities were evaluated by molecular docking.

**Results:**

96 DEGs with HIBD were identified in our result, including 89 up-regulated genes and 7 down-regulated genes. GO and KEGG results indicated that these DEGs were mostly enriched in Cytokine-cytokine receptor interaction, IL-17 signaling pathway and TNF signaling pathway. Using Cytoscape software and WGCNA-related modules, we identified three hub genes—*Tnf, IL1B*, and *Tlr2*—which were further validated in other transcriptomic datasets, all showing significant differential expression. Random forest analysis demonstrated that these three hub genes had AUC values > 0.75, indicating strong diagnostic performance. Immune infiltration analysis revealed that, compared to the control group, the HIBD group exhibited higher levels of innate immune cells (e.g., macrophages, M0 cells, and dendritic cells) and adaptive immune cells (e.g., CD8 naïve T cells, CD4 follicular helper T cells, and Th1 cells). The ssGSEA algorithm results indicated differences in 25 types of immune cells and 10 immune functions. The hub genes were also validated finally.

**Conclusion:**

*Tnf, Il1b* and *Tlr2* may be potential hub pyroptosis-related genes for HIBD. The results of this study could improve the understanding of the mechanisms underlying pyroptosis in HIBD.

## 1 Introduction

Neonatal hypoxic-ischemic encephalopathy (HIE), resulting from perinatal asphyxia, represents a significant cause of neonatal morbidity and mortality worldwide (1). The global incidence of HIE is approximately 2 per 1,000 live births, with even higher rates observed in developing country (2–4). Despite remarkable progress in perinatal medical technology in recent years, HIE continues to be a prevalent critical condition in neonatal intensive care units (NICUs). Its high mortality and disability rates impose a significant burden on affected families and society as a whole. Therapeutic hypothermia (TH) currently stands as the sole approved therapeutic intervention for hypoxic-ischemic brain damage (HIBD) (5). However, about 40% of HIE children receiving TH therapy still have audio-visual and motor behavioral impairments, such as total developmental delay and cerebral palsy (6, 7). Consequently, there is an urgent need to explore novel and effective neuroprotective strategies to improve outcomes for HIE patients.

The pathogenesis of HIBD is complex and involves the interplay of multiple pathophysiological processes (8, 9). Hypoxic-ischemic events lead to insufficient oxygen and energy supply to brain tissues, triggering a cascade of reactions, including energy metabolism failure, excitotoxicity, oxidative stress, inflammatory responses, and cell apoptosis. These processes collectively result in damage or even death of neurons and glial cells, ultimately leading to brain dysfunction. Pyroptosis is a recently identified form of programmed cell death characterized by its pronounced inflammatory features (10, 11). It is primarily triggered by the activation of inflammasomes and mediated by caspase-1 or caspase-4/5/11. Unlike apoptosis, pyroptosis involves the rupture of the cell membrane and the release of large quantities of pro-inflammatory cytokines, such as IL-1b and IL-18, thereby eliciting a robust inflammatory response. IL-1b (also known as IL1B) is a pro-inflammatory cytokine that plays a critical role in immune responses and inflammatory processes. The TNF superfamily (TNFSF) has 19 ligands and 29 receptors, serving as crucial mediators and regulators in human immuned inflammatory responses. A growing body of evidence indicates that tumor necrosis factor (TNF) is a potent proinflammatory cytokine implicated in the pathogenesis of various brain injury-related disorders. In addition, *Tlr2* is a member of the Toll-like receptor (TLR) family, which plays a pivotal role in the innate immune system. Currently, research on the role of Tlr2 in the HIBD model remains limited.

A growing body of research has reported that pyroptosis is associated with the pathophysiological processes of various neurological diseases, including ischemia–reperfusion injury post-stroke (12), Alzheimer’s disease (13), multiple sclerosis (14, 15) neonatal HIE (16) and other disease (17). Zheng et al. (18)demonstrated that diallyl disulfide can reduce hypoxic-ischemic (HI) injury by inhibiting the NLRP3-mediated pyroptosis signaling pathway. Overexpression of NLRP3 was shown to reverse this protective effect. Similarly, Tao et al. (19)reported that echinatin alleviates pyroptosis and inflammation in HIBD model by using TLR4/NF-kB pathway. The role and signaling pathways of pyroptosis in the pathogenesis of HIBD remain unclear. Although transcriptomic and genomic data have elucidated various aspects of cellular dynamics during pyroptosis, information on how pyroptosis operates in HIBD remains limited. Therefore, investigating the genomic changes that support pyroptosis and its involvement in HIBD can provide new insights for developing effective therapeutic strategies.

**Figure 1:**
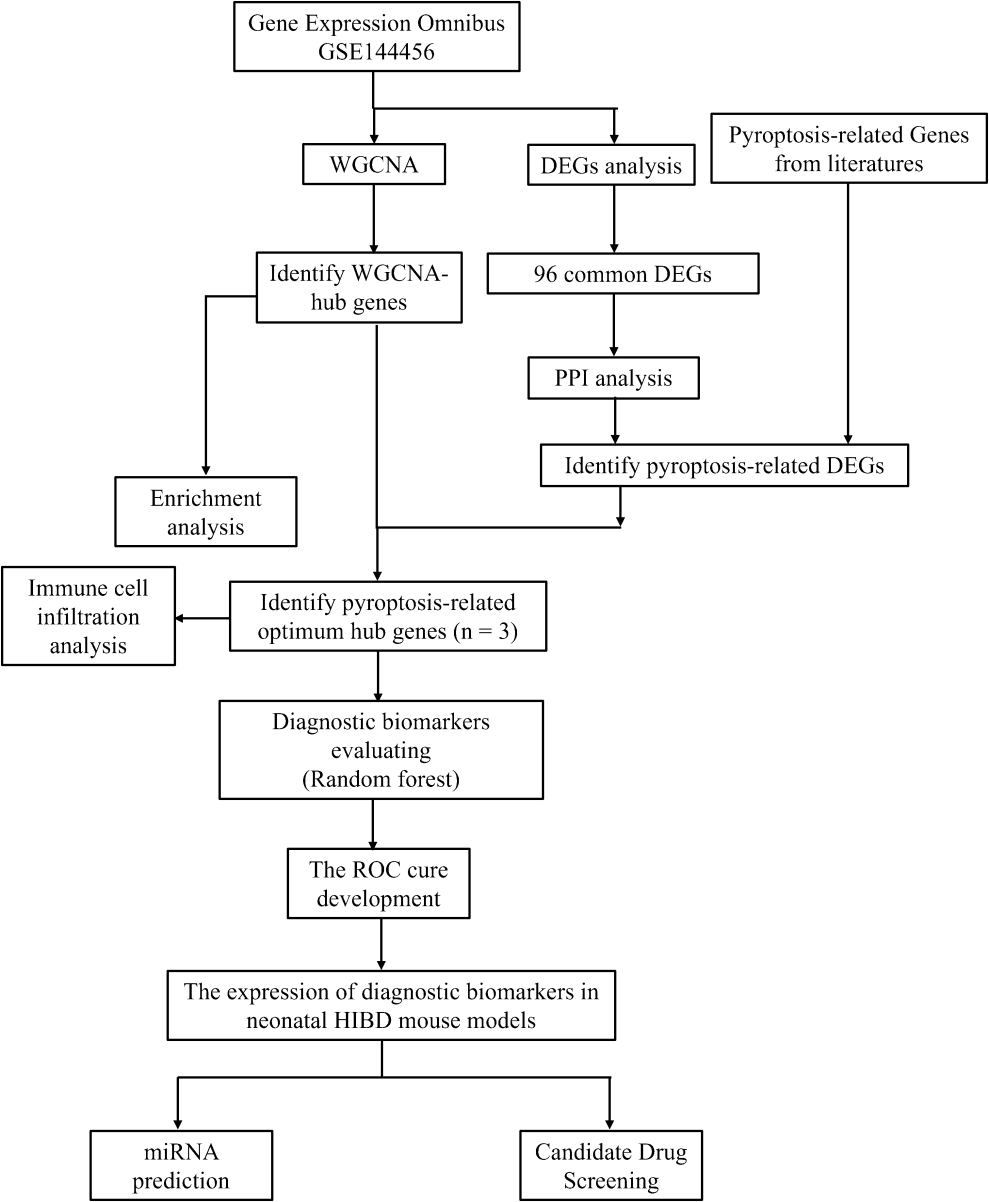
Flowchart in this study.

With the advancements in genomic microarray and high-throughput sequencing technologies, bioinformatics analysis has become an increasingly popular and powerful tool for exploring brain-related mechanisms and disorders. Currently, there is a lack of bioinformatics-based research on the mechanistic roles of pyroptosis-related genes (PRGs) in hypoxic-ischemic brain damage (HIBD). Therefore, this study aims to explore the molecular mechanisms of pyroptosis in HIBD through bioinformatics analysis. We identified core PRGs and investigated their relationship with immune cell infiltration. Additionally, we validated the expression patterns of these core immune-related genes using transcriptome sequencing and animal experiments (**Figure 1**). Collectively, these findings provide valuable insights for developing more effective therapeutic strategies.

## 2 Materials and methods

### 2.1 Data acquisition and processing

As illustrated in **Supplementary Table 1**, gene expression profiling dataset (GSE144456) related to hypoxic-ischemic brain injury (HIBD), was retrieved from the Gene Expression Omnibus (GEO) database (https://www.ncbi.nlm.nih.gov/geo/). A comprehensive list of 176 pyroptosis-related genes, which were identified from previous studies, is provided in **Supplementary Table 2**.

### 2.2 Differential expression analysis of genes and functional enrichment analysis

Differentially expressed genes (DEGs) in the GSE144456 dataset were identified using the limma package in R software (version 4.0.5, Auckland, New Zealand), with the significance thresholds set at a p-value < 0.05 and an absolute log2 fold change (|log2FC|) ≥ 0.5. We mapped the differentially expressed genes (DEGs) to each term in the Gene Ontology (GO) database (http://www.geneontology.org/) and calculated the number of DEGs associated with each term to obtain statistical data on the distribution of DEGs within specific GO functional categories. Subsequently, GO terms significantly enriched in the DEGs compared to the background gene set were identified using a hypergeometric test. Genes in organisms interact and coordinate to perform specific biological functions, and pathway-based analysis provides a systematic approach to elucidating the functional roles of individual genes in biological processes. In this study, the enrichment levels of DEGs in KEGG pathways (http://www.genome.jp/kegg/) were determined using the KOBAS (version 3.0) software. Pathways with a p-value < 0.05 were considered significantly enriched.

### 2.3 Protein–protein interaction network analysis

The PPI network was constructed by importing genes into the STRING database (www.STRING-db.org), a tool for retrieving protein-protein interactions, with interactions having a combined score > 0.5 being used for network construction. The network was visualized using Cytoscape (version 3.7.2), and genes within the network were ranked based on their degree centrality using the cytoHubba plugin.

### 2.4 Weight gene co-expression network analysis

To further investigate the potential roles of differentially expressed genes (DEGs) in hypoxic-ischemic brain damage (HIBD), we employed the R package “WGCNA” [10] to identify co-expression gene modules with high biological significance and to explore the relationship between gene networks and disease pathogenesis. All genes in the GSE144456 dataset were analyzed using WGCNA after removing missing values and duplicates. The optimal soft threshold was determined by setting R²=0.85, which enabled the identification of co-expression modules and key genes associated with HIBD. Subsequently, the adjacency matrix was converted into a topological overlap matrix (TOM), and modules were detected through hierarchical clustering with a minimum module size of 30 (minModuleSize=30). Specifically, module eigengenes (MEs) and modules were calculated independently and subjected to hierarchical clustering. The module eigengenes (MEs) and module membership (MM) values were used to identify fundamental modules related to HIBD. Finally, key modules were determined by analyzing the correlation between the expression data and module eigengenes. For further refinement, module genes exhibiting the highest gene significance values were intersected with pyroptosis-related hub genes.

### 2.5 Immune cell infiltration analysis

Immune cell infiltration analysis is a method used to quantify the abundance and spatial distribution of different immune cell types in pathological tissues, aiming to reveal the characteristics of the immune microenvironment and its relationship with disease progression. CIBERSORT is a widely used deconvolution algorithm based on gene expression data. In this study, mRNA expression profiles of cortical tissues from the HIBD group and the control group were analyzed, and the relative proportions of 22 immune cell types in both groups were evaluated using the CIBERSORT algorithm. A histogram illustrating the proportion of immune cells, a box plot depicting immune cell expression levels, and a box plot showing the results of differential immune cell analysis were generated. Subsequent analyses retained only statistically significant data, with a CIBERSORT *p*-value < 0.05. Additionally, this study performed correlation analyses to evaluate the relationship between hub pyroptosis-related genes and immune infiltration in HIBD. Single-sample gene set enrichment analysis (ssGSEA), an extension of Gene Set Enrichment Analysis (GSEA), has been widely employed in immune infiltration-related bioinformatics studies (20). Enrichment scores for 28 immune cell subsets and 13 immune functional pathways were computed in both normal and hypoxic-ischemic brain damage (HIBD) samples using the GSVA R package, with results visualized via the Vioplot R package. Subsequently, Spearman’s rank correlation analysis was performed to assess associations between hub pyroptosis-related genes sand immune cell infiltration/functional profiles.

### 2.6 Machine learning

To better evaluate the diagnostic efficacy of hub pyroptosis-related genes and provide a robust computational biology foundation for subsequent targeted therapy, we further employed the random forest approach. A Random Forest classification model was constructed, and its performance was assessed using a confusion matrix, accuracy, sensitivity, specificity, and ROC-AUC values.

### 2.7 Animals and the hypoxic-ischemic brain damage model

In this study, all experimental procedures were approved by the Institutional Animal Care and Use Committee of The Third Affiliated Hospital of Zhengzhou University. Male and female C57BL/6 mice were obtained from the Laboratory Animal Center [Henan, China]. The pups, along with their mothers, were housed under controlled environmental conditions, including an ambient temperature of 22 ± 2°C, relative humidity of 56 ± 5%, and a 12-hour light/dark cycle. As previously described, on postnatal day 9, C57BL/6 mice (average body weight: 5 ± 0.3g) were anesthetized with isoflurane (3.5% for induction and 1.5% to 2% for maintenance). A midline incision (approximately 1cm in length) was made in the neck to expose the right common carotid artery (CCA). The CCA was double-ligated using 6–0 surgical silk sutures, and a specialized micro-clamp was used to create an incision between the two ligation points. The surgical procedure for each pup was completed within approximately 5 minutes. After the procedure, the pups were allowed to recover until they resumed normal breathing and were then returned to their mothers. Following a 1-hour recovery period, the mice were placed in a hypoxic chamber maintained at 37°C with a gas mixture of 10% oxygen and 90% nitrogen. The sham group underwent the same procedures except for arterial ligation and hypoxia exposure.

### 2.8 TTC and H&E staining

In order to ensure the efficient construction of HIBD model, TTC and HE staining techniques were used. TTC staining is a classical method for assessing cerebral infarction volume. According to previous literature reports (4, 21), TTC staining clearly demarcates the infarct core as pale unstained regions, while viable tissue stains deep red. The infarct volume ratio is calculated using the formula: Infarct volume percentage=(Contralateral hemisphere volume–Ipsilateral non-infarcted volume)/Contralateral hemisphere volume × 100%. H&E staining is one of the most commonly used histopathological staining methods for observing morphological changes in brain tissue after ischemic injury, including neuronal necrosis, inflammatory infiltration, and other pathological features. Based on previous studies (22–24), H&E staining was performed on mice 3 days after HIBD surgery. This method allows qualitative assessment of ischemic injury by distinguishing normal neurons from hypoxic-ischemic damaged neurons, as well as localization of the infarct core.

### 2.9 Quantitative real‐time polymerase chain reaction

Total RNA was extracted from the brain tissues of the ipsilateral HIBD or sham group using TRIzol reagent from Life Technologies, and then the optical density of the extracted RNA was measured. If the optical density was between 1.8 and 2.2, the sample was considered usable. 1 μg of the usable RNA was reverse-transcribed into 20 μL of cDNA. Then, 2 μL of the cDNA was amplified with specific primers and SYBR PCR Master. The reverse-transcription amplification kit was provided by Vazyme. GAPDH was used as an internal reference. The values of the target genes were normalized to the fold-change determined using the 2^−ΔΔCT^ method. The primer sequences used in this study are listed in **Table 1**.

**Table 1:** Primers used for qRT-PCR.

Gene	Forward primer	Reverse primer
β-actin	ACGGCCAGGTCATCACTATTG	AGAGGTCTTTACGGATGTCAACGT
Tnf	TATGGCTCAGGGTCCAACTC	GGAAAGCCCATTTGAGTCCT
Il1b	TGCCACCTTTGACAGTGATG	TGATGTGCTGCTGCGAGATT
Tlr2	CTCTTCAGCAAACGCTGTTCT	GGCGTCTCCCTCTATTGTATTG

### 2.10 Immunofluorescence

Tissue samples were collected at 24 hours post-HIBD for histological examination. Tissues were embedded in Optimal Cutting Temperature Compound (OCT, Fisher Scientific) and then sectioned into 15 μm slices. Sections were stained with Tlr2 (1:200, Abcam), Il1b (1:200, Abcam) and Tnf (1:200, Abcam) at 4°C overnight. Appropriate fluorescence conjugated secondary antibodies (Proteintech, China) were incubated for 1 h at room temperature. Nuclei were counterstained with DAPI. Immunofluorescence images were acquired on an Olympus IX83 microscope equipped with a 40× objective (NA 0.95).

### 2.11 Construction of pyroptosis -related mRNA-miRNA regulatory network

To explore the regulatory mechanisms of the optimal hub pyroptosis -related genes, an mRNA-miRNA regulatory network was constructed. The miRWalk3.0 online tool, a comprehensive database of miRNA target genes, was used to predict miRNAs potentially. This database includes miRNA target gene information for multiple species, such as mouse and rats. MiRWalk was used to predict miRNAs targeting the optimal hub pyroptosis -related genes. A miRNA-hub pyroptosis -related genes interaction network was visualized using Cytoscape software.

### 2.12 Molecular docking

Schrodinger2021 was employed to dock the Top5 drugs or small molecules screened from DSigDB to investigate the binding interactions between target proteins and ligand molecules (25, 26). Briefly, the crystal structures of target proteins were obtained from the Protein Data Bank (PDB) (2Z81, 2tnf, 8RYS). Before docking, protein structures were processed using the Protein Preparation Wizard in Schrodinger software. This involved steps such as removing all water molecules, adding hydrogen atoms, further optimizing the structure with the OPLS3 force field, and minimizing protein energy. The Sitemap module was used to generate docking boxes. Small molecules were obtained from Pubchem with SDF structures and underwent conformation minimization and energy minimization processing through the LigPrep module. Docking was carried out using the Glide module, and the Maestro module was used for result analysis and processing.

## 3 Results

### 3.1 Data acquisition and processing

We obtained hypoxic-ischemic brain damage (HIBD) related datasets from the Gene Expression Omnibus (GEO) database (http://www.ncbi.nlm.nih.gov/geo/). Datasets were selected according to the following criteria (1): the profile information should encompass both disease and control groups and (2) the datasets need to provide raw data for further analysis. Therefore, GEO dataset GSE144456 was selected for subsequent investigation. GSE144456 contained 24 HIBD samples and 8 control samples. For these datasets, GeneSymbol mapping was carried out based on their respective platforms. In instances of multiple matches, the average value was adopted. The final expression matrix was derived by normalizing using the log2(X + 1) method. During preprocessing, after the initial quality control assessment, the “normalizeBetweenArrays” function in the “limma” package was utilized to perform quantile normalization. This method adjusted the expression values such that each sample had an identical empirical distribution of expression values, thereby effectively minimizing technical variations between samples.

### 3.2 Identification of common DEGs and enrichment analysis

**Figure 2:**
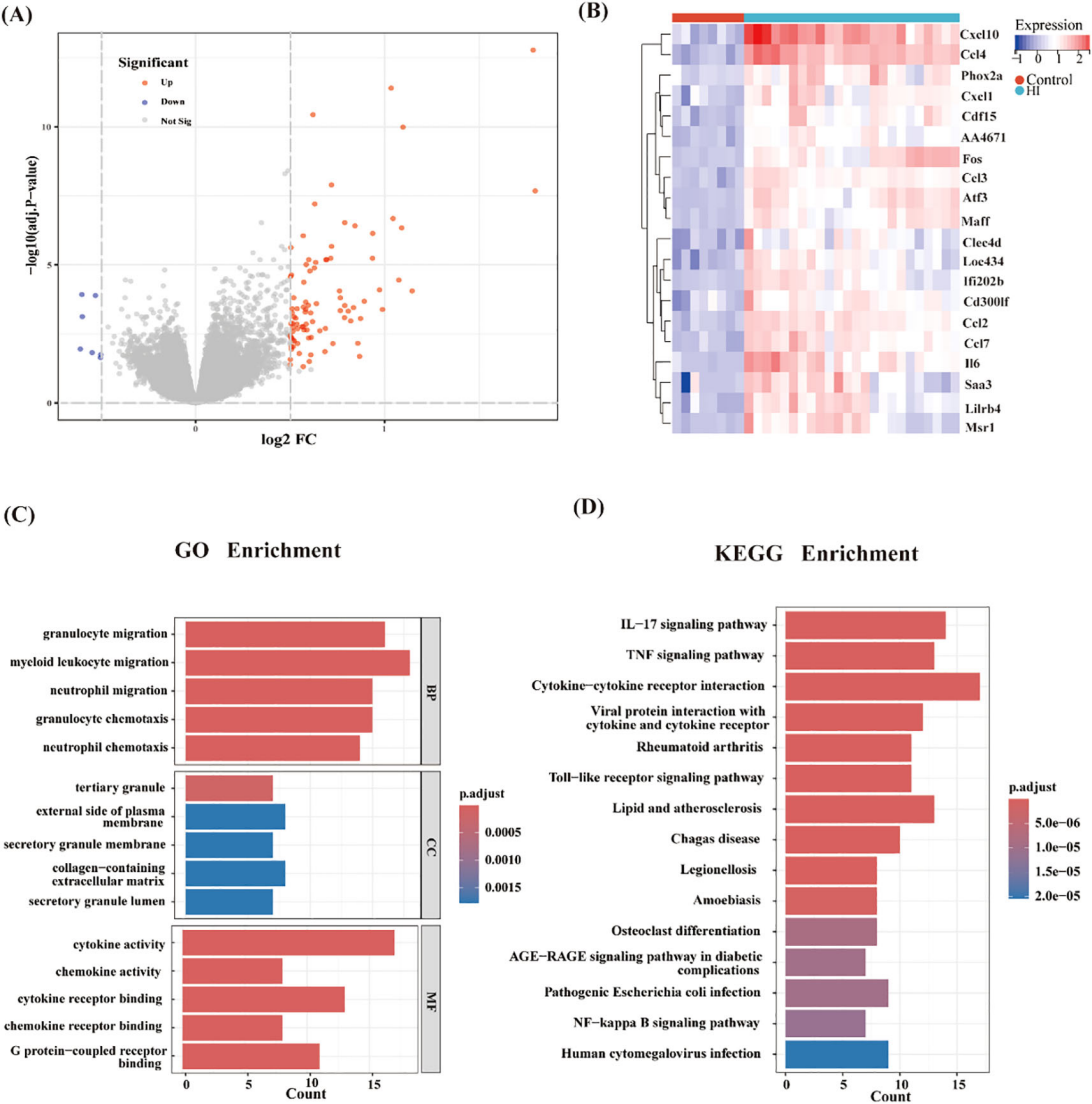
Identification of common DEGs in GSE144456. **(A)** Volcano plot of all DEGs in GSE144456, red indicates upregulated DEGs, and blue indicates downregulated DEGs. **(B)** A heatmap of the top 20 DEGs in GSE144456. **(C, D)** The enrichment analysis results of GO and KEGG pathway.

**Figure 3:**
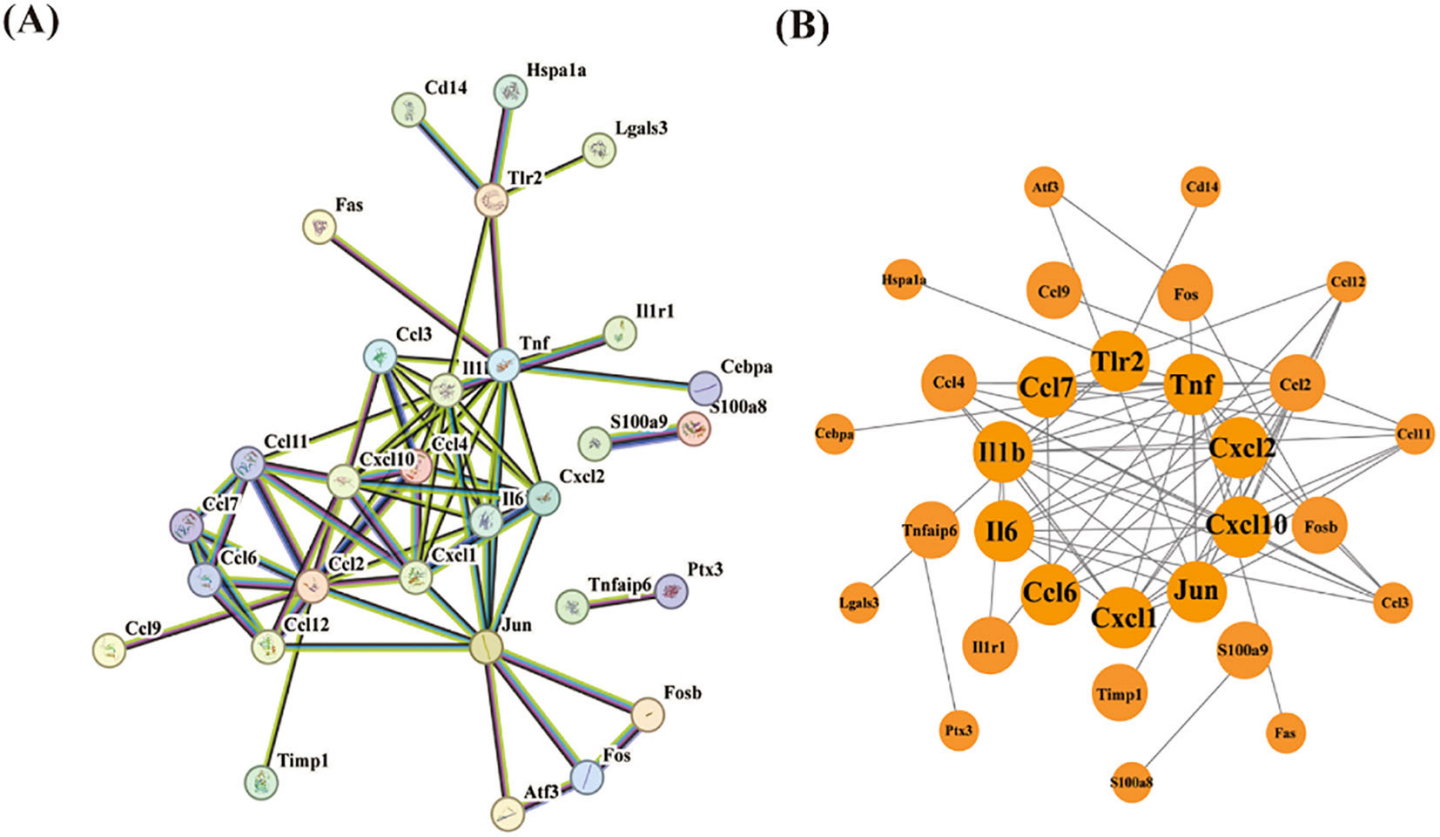
PPI network analysis of DEGs. **(A)** The PPI network of 30 DEGs was constructed by using String database. **(B)** First ten genes with the highest degree identified by Cytoscape software and CytoHubba.

Following quality control, normalization, and background adjustment of the GSE144456 dataset, we identified 96 significantly altered genes using thresholds of adjusted p-value < 0.05 and |log2 fold change| > 0.5. As illustrated in the volcano plot (**Figure 2A**), 89 genes were up-regulated and 7 genes were down-regulated under the experimental conditions. The heatmap illustrated the top 20 most differentially expressed genes (DEGs) (**Figure 2B**). Notably, in the GSE144456 dataset, all top 20 altered genes were up-regulated, implying that their high expression promotes HIBD development. To functionally characterize the 96 DEGs, Gene Ontology (GO) and Kyoto Encyclopedia of Genes and Genomes (KEGG) pathway enrichment analyses were performed to elucidate the biological functions. The GO results showed the top five biological processes (BP), cellular components (CC), and molecular functions (MF). These genes were mainly involved in biological processes like granulocyte migration, myeloid, leukocyte migration, granulocyte migration and neutrophil chemotaxis (**Figure 2C**). Regarding cellular components (CC), proteins encoded by these genes were mainly located on the tertiary granule, external side of plasma membrane. Secretory granule membrane, collagen-containing extracellular matrix and secretory granule lumen. And the molecular functions also displayed. The KEGG results indicated that these 96 genes were mainly distributed in signaling pathways like Cytokine-cytokine receptor interaction, IL-17 signaling pathway and TNF signaling pathway (**Figure 2D**). In addition, to further screen for significant genes, we input the 96 candidate DEGs into the String database and removed independent genes (**Figure 3A**). Then, Cytoscape software was used to display proteins interacting with each other according to their degrees. To further screen out hub genes, the MCC algorithm in the Cytohubba plugin was used to determine the top ten genes in the above-mentioned PPI network (**Figure 3B**). Eventually, Tlr2, Tnf, Cxcl2, Cxcl10, Jun, Cxcl1, Ccl6, Il6, Il1b and Ccl7 were identified as candidate hub genes for HIBD.

### 3.3 Weighted gene co-expression network analysis

**Figure 4:**
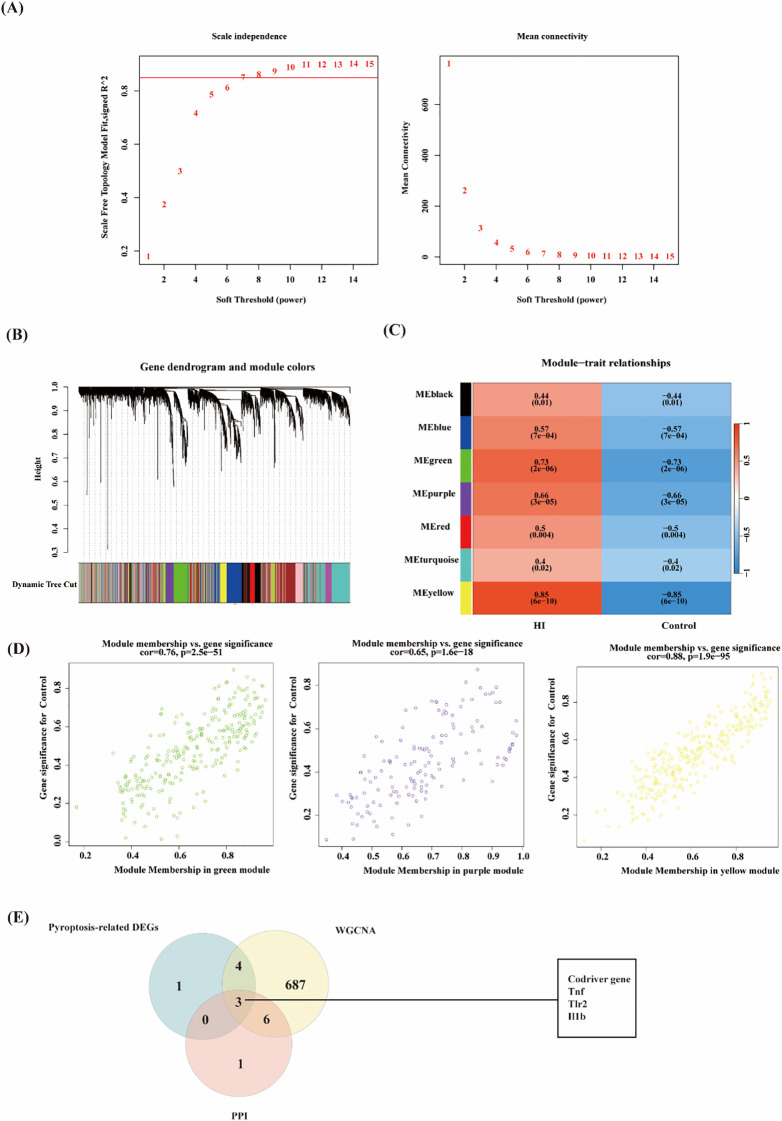
WGCNA analysis of the significant modules in GSE144456 and evaluation of pyroptosis-related Gens. **(A)** The selection of soft threshold in GSE144456. **(b)** Gene cluster dendrogram for GSE144456 by dynamic tree cut algorithm. **(C)** Heatmap of the association between modules and clinical traits in GSE144456. **(D)** Correlation Plot of Module Membership and Gene Significance in Green, Purple, and Yellow Modules. **(E)** Venn diagram of overlapping genes among the co-expression modules identified by WGCNA, pyroptosis-related gene DEGs and PPI genes.

In this study, to further explore the key genes in HIBD, the gene co-expression network of the mRNA dataset was constructed by utilizing the R package of WGCNA. Based on the evaluation of scale-free topology fit (scale independence) and mean network connectivity, an optimal soft-thresholding power (β) of 7 was selected (**Figure 4A**) to ensure biologically meaningful network construction while preserving moderate connection density. In total, 12 modules were obtained (**Figure 4B**). The clustering of module eigengenes showed in **Supplementary Figure**. Moreover, 8 modules were investigated through module-trait analysis, revealing statistically significant correlations (*p* < 0.05) as shown in **Figure 4C**. These data showed that the purple (142 genes, r=0.66, *p*=3e-05), green (265 genes, r=0.73, *p*=2e-06) and yellow (290 genes, r=0.85, *p*=6e-10) exploring the most positive correlation between HIBD and gene modules. Furthermore, we investigated a strong association between module membership and gene significance in the green (r=0.76, *p* =2.5e−51), purple (r=0. 65, *p* =1.6e−18) and yellow (r=0.88, *p* =1.9e−95), respectively (**Figure 4D**). Pyroptosis-related DEGs among HIBD-associated genes including Tnf, Tlr2, Neat1, Cebpa, Il1r1, Lgals3, S100a9 and Il1b. All these genes are upregulated genes. Venn diagram analysis revealed seven overlapping genes among the co-expression modules identified by WGCNA, HIBD-associated DEGs, and pyroptosis-related gene sets, suggesting potential mechanistic links between these molecular networks. These three optimal hub pyroptosis-related DEGs (PRDEGs) were selected for further analysis (**Figure 4E**).

### 3.4 Immune cell infiltration and its correlation with optimal hub PRDEGs

**Figure 5:**
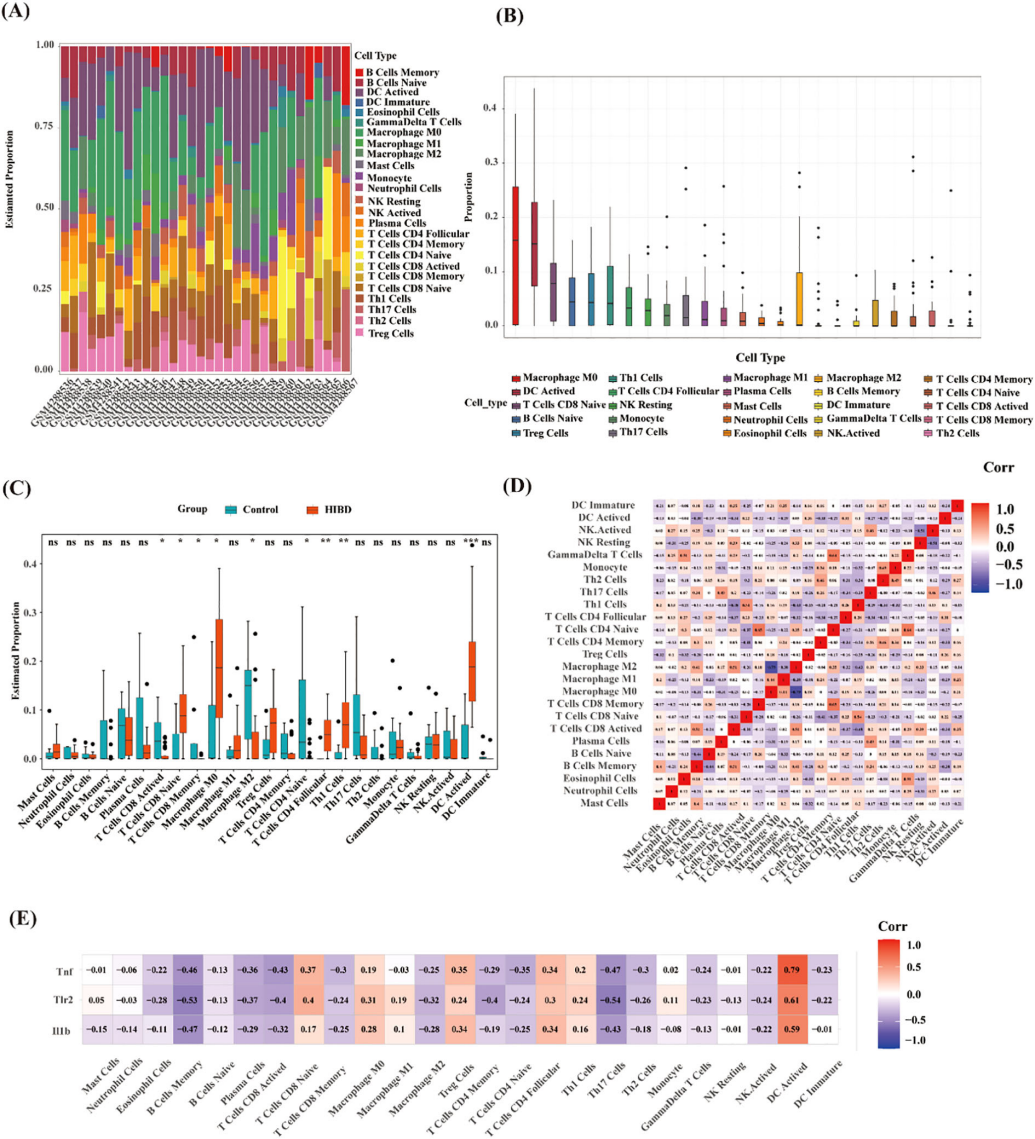
Immune cell infiltration analysis. **(A)** The proportions of the 22 detected immune cells in each sample; **(B)** Infiltrating abundances of immune cell infiltration; **(C)** Differences of immune cells between HIBD model and control group. **(D)** Correlation analysis between infiltrated immune cells; **(E)** Relationship between the hub pytoptosis-related genes and immune infiltration. ns (*p* ≥ 0.05), *(*p* < 0.05), **(*p* ≤ 0.01), ***(*p* ≤ 0.001).

Based on previous enrichment results, the optimum pyroptosis-related genes in HIBD were found to be primarily associated with immune-related pathways. Using the CIBERSORT algorithm, the proportions and infiltration abundance of 22 immune cell types detected in each sample were visualized as a histogram (**Figure 5A**) and boxplot (**Figure 5B**), respectively. Significant differences in immune cell profiles were observed between the HIBD group and the control group. Compared to the control group, HIBD group showed higher levels of CD8+ T naïve cell, Macrophage M0 cell, CD4+ T cell follicular, Th1 cells and DC activated cell. In addition, the levels of plasma cells, CD8+ T activated cells, CD8+ T memory cell and CD4+ T naïve cells were lower in HIBD group (**Figure 5C**). The results of immune cell variation analysis are presented in **Figure 5D**. Gene-immune cell correlation analysis revealed strong positive correlations between the expression of Tnf, Tlr2 and Il1b (**Figure 5E**).

**Figure 6:**
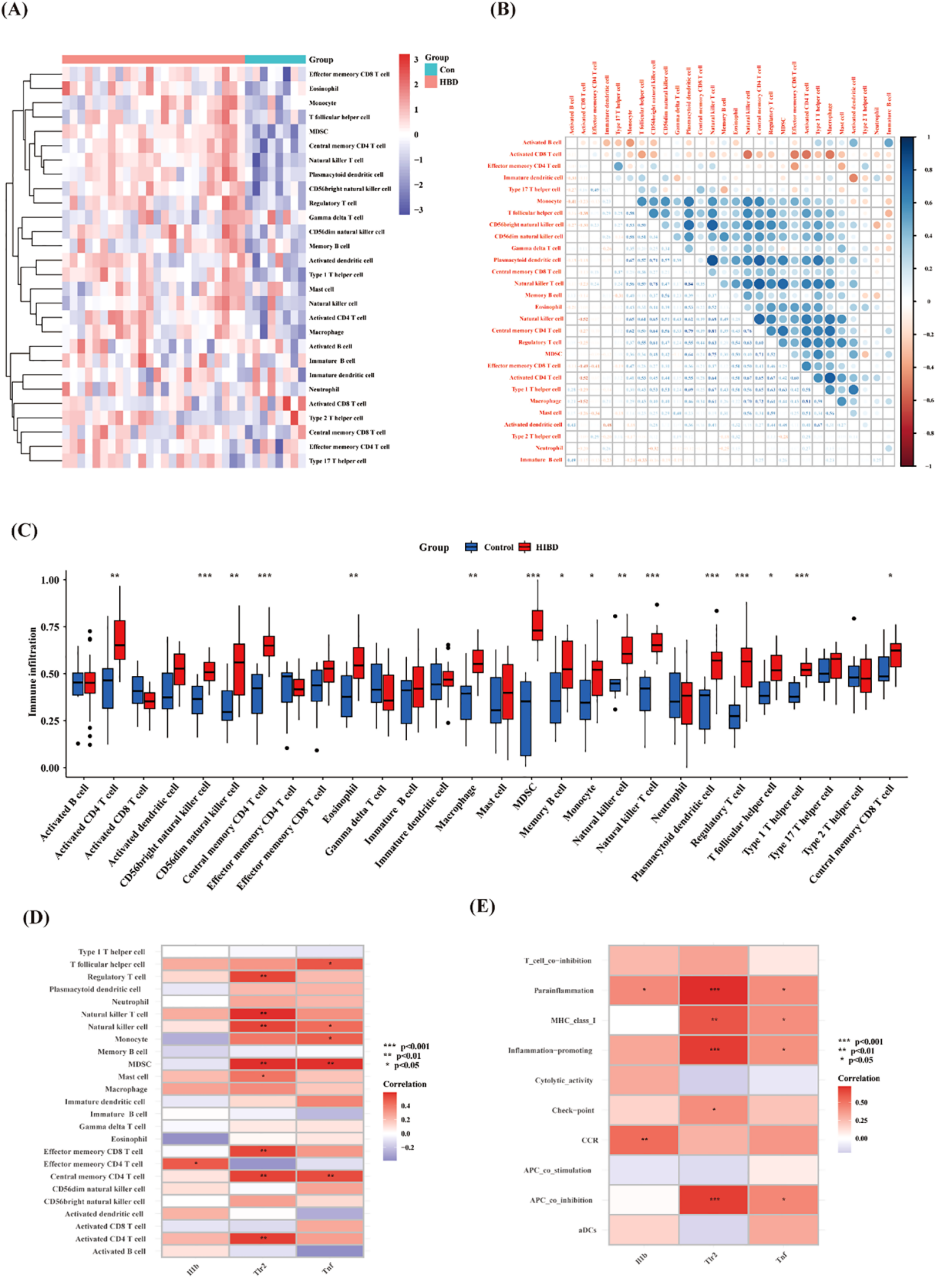
ssGSEA immune infiltration. **(A)** Heat map of the differences in the distribution of 28 immune cells in each sample; **(B)** Correlation analysis between infiltrated immune cells; **(C)** Violin plots of differences in the infiltration of 28 immune cells between control and HIBD group; **(D, E)** Correlation analysis of hub pyroptosis-related genes with 28 immune cells and 13 immune functions. Note: ns (*p* ≥ 0.05), *(*p* < 0.05), **(*p* ≤ 0.01), ***(*p* ≤ 0.001).

To further explore the correlation between samples and immune status, we evaluated the enrichment scores of 28 immune cell types using ssGSEA. The results showed that there were significant differences in the levels of immune infiltration between the control and HIBD groups (**Figures 6A, C**). For example, CD56bright natural killer cell, Central memory CD4 T cell, MDSC, natural killer T cell, plasmacytoid dendritic cell, regulatory T cell, and type 1 T helper cell were significantly increased in HIBD groups. There is a significant correlation among most immune cells (**Figure 6B**). In addition, Il1b, Tlr2 and Tnf correlated well with 25 types of immune cells and ten types of immune functions (**Figures 6D, E**). For example, Il1b was positively correlated with Effector memory CD4 T cell. Tlr2 was positively correlated with Regulatory T cell, Natural killer T cell, Natural killer cell, MDSC, Mast cell, Effector memory CD8 T cell, Central memory CD4 T cell and Activated CD4 T cell. Tnf was positively correlated with T follicular helper cell, Natural killer T cell, Monocyte, MDSC and Central memory CD4 T cell.

### 3.5 Evaluation of diagnostic effectiveness using machine learning

**Figure 7:**
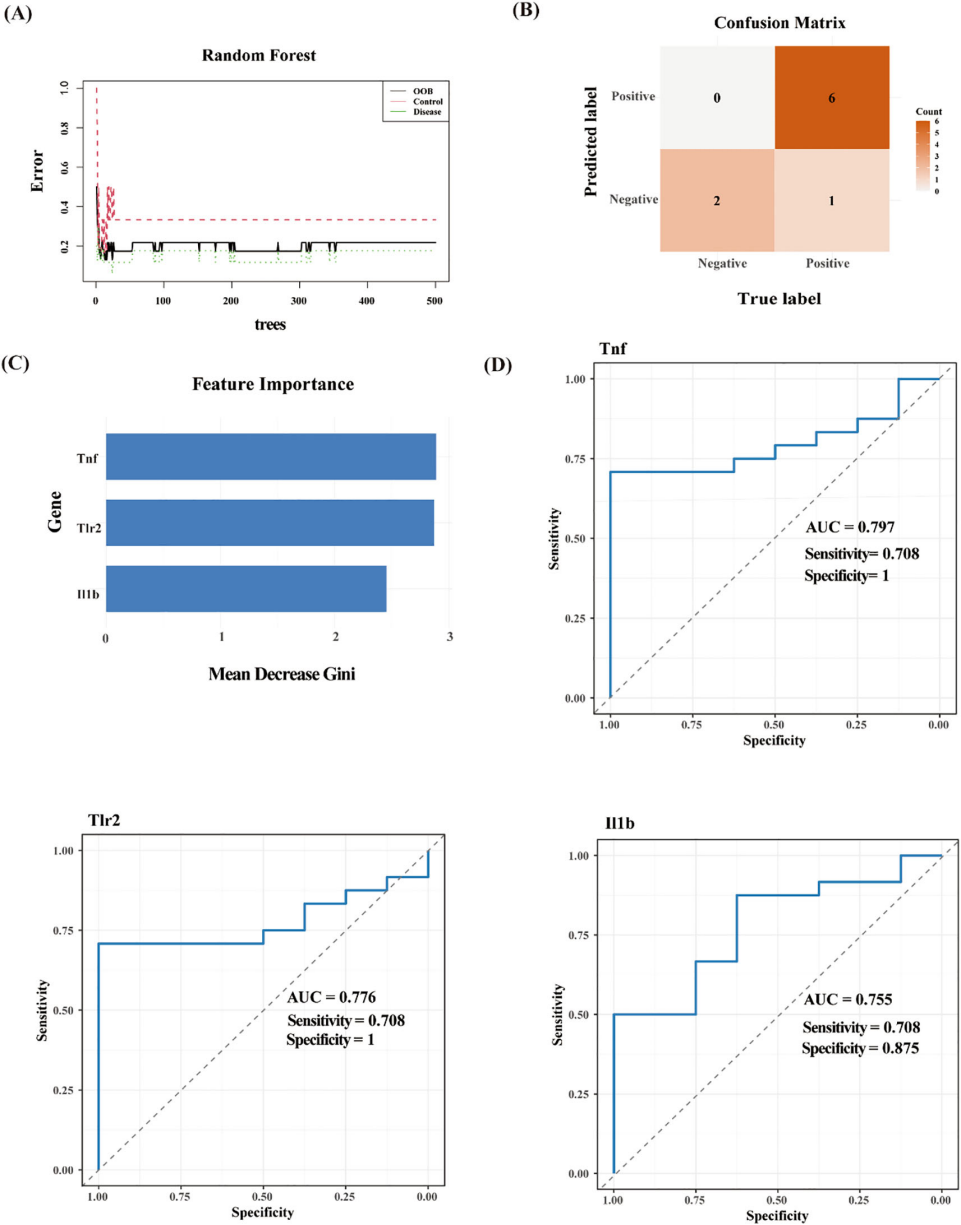
Evaluation of diagnostic effectiveness by Random Forest analysis. **(A)** Based on the random forest algorithm, the correlation between the total number of trees and the error rate in the HIBD dataset. **(B)** The Confusion Matrix diagram. **(C)** The ranking of gene relative importance scores. **(D)** ROC curves of the Tnf, Il1b and Tlr2.

To further evaluate the diagnostic effectiveness of hub pyroptosis-related genes, we applied the random forest machine learning algorithm. The constructed model revealed these three genes demonstrated significant diagnostic potential, with variable importance measure scores exceeding the critical threshold of 1 (**Figures 7A, C**).

The Confusion Matrix is an important tool for evaluating the performance of classification models, as shown in **Figure 7B**. And the F1-score was 0.9231 (demonstrating strong precision-recall balance), the accuracy was 0.8889 (indicating robust overall classification performance). And the AUCs of these three genes all > 0.75 (**Figure 7D**). This machine learning approach effectively prioritized these candidate genes based on their relative contributions to distinguishing between experimental groups, suggesting their potential utility as molecular biomarkers for HIBD detection.

### 3.6 Validation of hub pyroptosis-related genes

**Figure 8:**
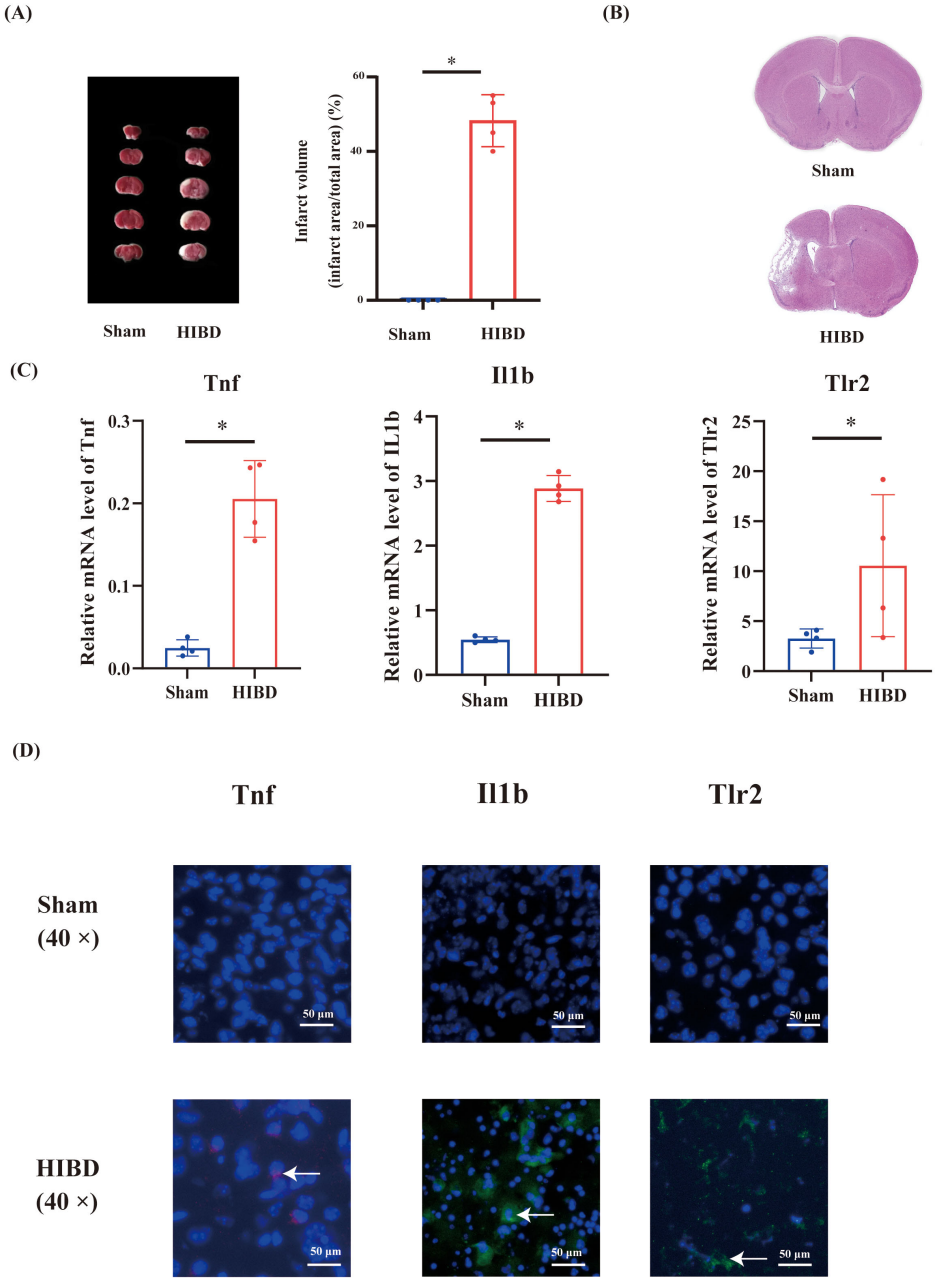
Validation of hub pyroptosis-related genes in HIBD mice. **(A)** The image of TTC between HIBD and sham group. **(B)** The image of H&E staining. **(C)** The expression level of Tnf, Il1b and Tlr2 using qRT-PCR. **(D)** Representative immunofluorescence images showing the expression patterns of Tnf, Il1b, and Tlr2 genes in both groups. * p < 0.05.

To validate the reliability of the GSE144456 dataset analysis, we performed qRT-PCR and immunofluorescence assays. Firstly, in order to validate the efficacy of the neonatal mouse HIBD model, we initially performed TTC and HE staining analyses (**Figures 8A, B**). The results demonstrated significant infarct foci in the HIBD group compared with the sham group. Furthermore, HE staining provided qualitative assessment of the cerebral damage in HIBD mice. Subsequently, we verified the expression levels of hub pyroptosis-related genes using qRT-PCR, which revealed statistically significant differences (p < 0.05, **Figure 8C**). Moreover, immunofluorescence staining was employed to evaluate the spatial expression patterns of these hub genes in peri-infarct regions (**Figure 8D**). Finally, external validation using independent transcriptomic datasets yielded consistent results with our findings.

### miRNA prediction

**Figure 9:**
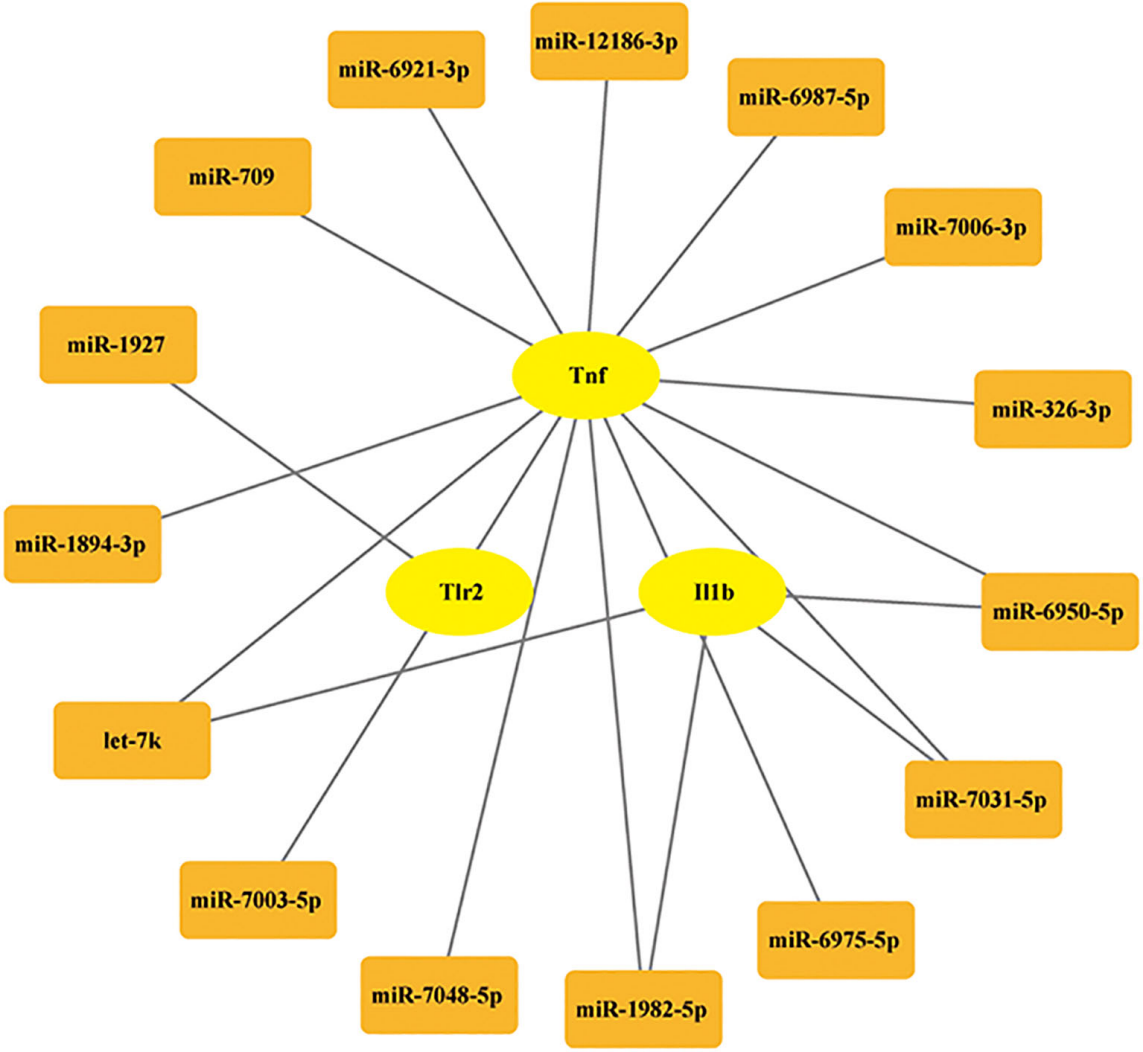
The regulatory network of the hub PRDEGs-miRNA.

miRNAs are a class of endogenously expressed small non-coding RNAs that post-transcriptionally regulate gene expression through targeted messenger RNA (mRNA) degradation or translational repression. Previous studies indicated that miRNAs play crucial regulatory roles in multiple pathophysiological processes relevant to neonatal hypoxic-ischemic encephalopathy (27, 28). Therefore, in this study, we also conducted predictions of miRNA target genes. The miRNAs associated with the hub PRDEGs were identified using the miRNA prediction platform. The prediction results were filtered based on the following stringent criteria: accessibility probability (p) < 0.05, binding score > 0.95, and targeting of the 3’ UTR region. As shown in **Figure 8**, we constructed a comprehensive gene-miRNA regulatory network comprising 15 miRNAs. These miRNAs were selected based on their network degree (Degree ≥ 4, **Figure 9**), suggesting their pivotal regulatory roles in modulating multiple hub PRDEGs and underscoring their central position in the regulatory network.

### 3.7 Candidate drug screening based on hub genes

**Figure 10:**
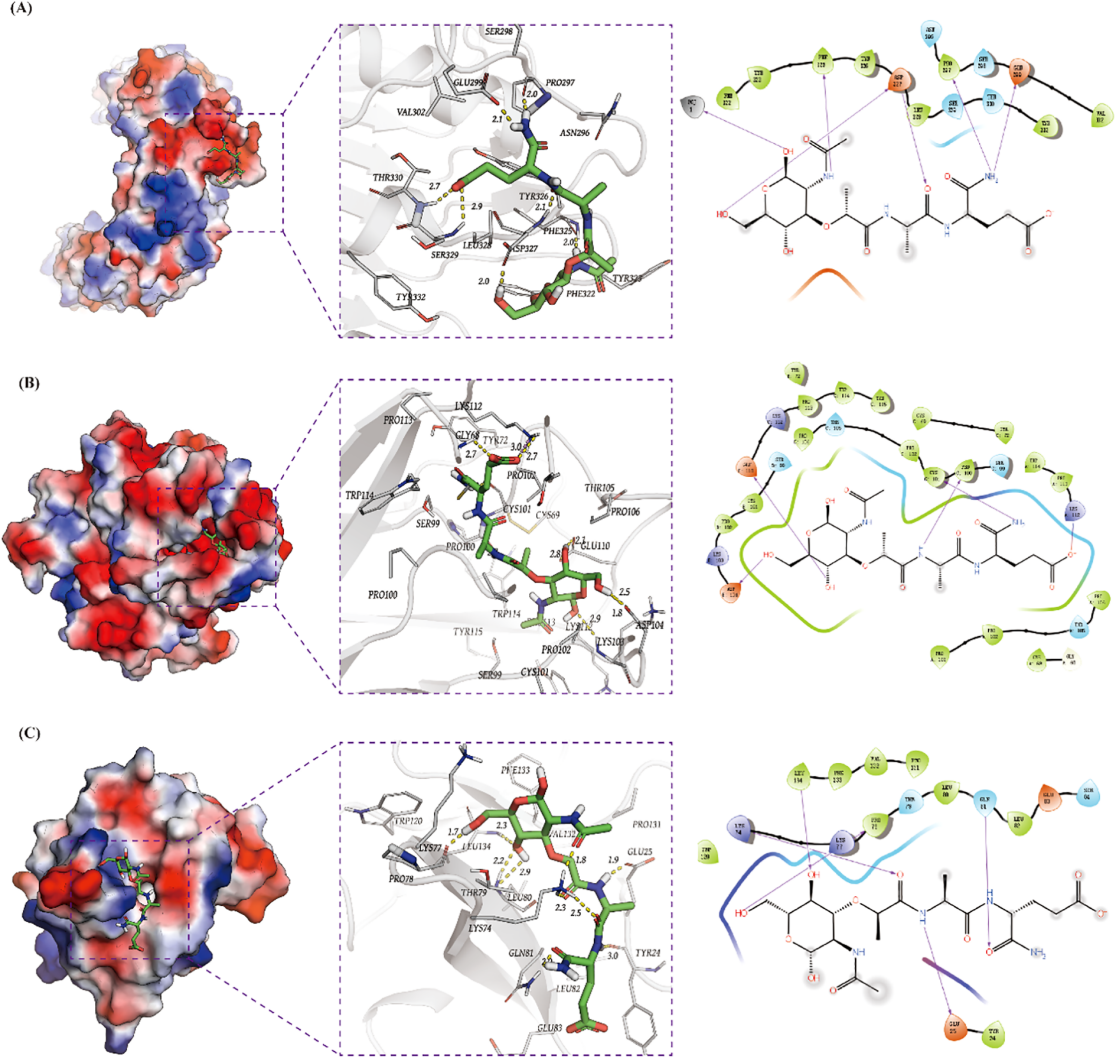
Screening of potential candidate drugs for treating HIBD base on TLR2, TNF-α and IL1B. **(A–C)** 3D and 2D structures of the highest score molecule muramyl dipeptide for TLR2, TNF-αand IL1B.

Through systematic screening of the DSigDB database in Enrichr, we identified 1,130 potential drugs or small molecules targeting the three hub genes (TNF-α, IL1B, and TLR2) based on stringent criteria (P < 0.05 and favorable binding scores with core hub genes; **Table 2**). Among these candidates, muramyl dipeptide emerged as the top-scoring molecule and was subsequently selected for molecular docking studies with the identified hub genes. The docking analysis revealed a robust interaction between muramyl dipeptide and TLR2, with a docking score of -4.48 (**Figure 10A**). Structural examination demonstrated that muramyl dipeptide binds specifically to the ligand-binding pocket of TLR2, which is formed by key residues (Asn296, Pro297, Ser298, Glu299, Val302, Phe322, Tyr323, Phe325, Tyr326, Asp327, Leu328, Ser329, Thr330, and Tyr332). The binding mechanism involves multiple hydrogen bond interactions (1): The hydroxyl and carbonyl groups of muramyl dipeptide form hydrogen bonds with the side chain of Asp327 (2). The amino and imino groups of MDP engage in hydrogen bonding with the side chain of Glu299 and the backbone imino groups of Pro297 and Phe325, respectively. The results showed that the docking score between TNF-α and muramyl dipeptide was -6.47 (**Figure 10B**). Muramyl dipeptide selectively binds to the ligand-binding pocket of tumor necrosis factor-α (TNF-α), which is collectively constituted by the amino acid residues Lys112, Pro113, Gly68, Tyr72, Pro102, Thr105, Trp114, Cys101, Pro106, Cys69, Ser99, Glu110, Pro100, Asp104, Tyr115, and Lys103 (note: Lys112 is listed twice in the original sequence, retained here as provided). The binding mechanism involves multiple hydrogen bond interactions: the hydroxyl group forms hydrogen bonds with the side chains of Asp104 and Glu110; the amino and imino groups engage in hydrogen bonding with the side chain of Cys101; and the carboxyl group interacts via hydrogen bonds with the side chain of Lys112. The results showed that the docking score between IL1b and muramyl dipeptide was -6.40 (**Figure 10C**). Muramyl dipeptide selectively binds to the ligand-binding pocket of interleukin-1β (IL-1β), which is collectively formed by the amino acid residues Trp120, Pro131, Val132, Leu134, Lys77, Glu25, Pro78, Thr79, Leu80, Lys74, Gln81, Tyr24, Leu82, Glu83, and Phe133. The binding interaction involves specific hydrogen bond formations: the hydroxyl group forms hydrogen bonds with the side chains of Lys77 and Leu134; the carbonyl group engages in hydrogen bonding with the side chains of Glu25 and Gln81; and the amino group interacts via hydrogen bonds with the side chain of Glu25. These results indicate that muramyl dipeptide have the potential to become therapeutic drugs targeting the three hub genes TNF-α, IL1b, and TLR2, thereby synergistically controlling the occurrence and development of HIBD.

**Table 2:** The top 10 significant P-values for DsigDB.

Term	*p*-value	Overlap_genes
Muramyl Dipeptide CTD 00005307	1.15490e-09	IL1B, TNF, TLR2
Peptidoglycan CTD 00006490	3.371144e-09	IL1B, TNF, TLR2
Uric acid BOSS	6.32687e-09	IL1B, TNF, TLR2
N-NITROSODIETHYLAMINE BOSS	1.967625e-08	IL1B, TNF, TLR2
Sodium sulfate BOSS	3.276027e-08	IL1B, TNF, TLR2
Sodium dodecyl sulfate CTD 00006753	4.473060e-08	IL1B, TNF, TLR2
Hydrocortisone CTD 00006117	1.105831e-07	IL1B, TNF, TLR2
ACMC 20mvek CTD 00002629	1.212785e-07	IL1B, TNF, TLR2
D Sorbitol BOSS	1.212785e-07	IL1B, TNF, TLR2
Titanium BOSS	1.212785e-07	IL1B, TNF, TLR2

## 4 Discussion

Neonatal hypoxic-ischemic encephalopathy (HIE) is a severe brain injury resulting from perinatal hypoxia and/or inadequate cerebral perfusion. This condition can trigger neuronal death and neurological dysfunction, making it one of the most prevalent forms of neonatal brain injury and a leading cause of infant mortality and long-term neurological sequelae. In recent years, numerous preclinical studies have demonstrated that mitigating pyroptosis confers neuroprotective effects in rodent models of hypoxic-ischemic brain damage (HIBD) (18, 29, 30). However, the precise role of pyroptosis-related genes in HIBD remains incompletely understood. Therefore, our study aims to elucidate the underlying molecular mechanisms of pyroptosis in HIBD.

In this study, we first screened brain tissue samples from HIBD and control groups in mice using the GEO database. Differential gene expression analysis identified 96 significantly differentially expressed genes (DEGs), followed by functional enrichment analysis. GO and KEGG enrichment analyses revealed that these genes were primarily enriched in receptor complex components and involved in immune cell activation, and cytokine-cytokine receptor interaction. Preliminary studies suggest that axonal injury is a significant pathological process in HIBD and may be associated with long-term neurological dysfunction in surviving children with HIBD (31, 32). Recent research have also reported that the use of various drugs to promote axonal repair exerts neuroprotective effects against brain injury in HIBD (33, 34). Reimer et al. reported (35) that myelinated axonal injury is associated with mild cerebral hypoperfusion, and further microarray analysis demonstrated that the key biological pathways involved were cytokine-cytokine receptor interactions and inflammatory responses. This finding is consistent with our experimental observations. These results indicate that cytokine-cytokine interaction plays an important role in HIBD model.

PPI network analysis is an integrative research methodology that combines transcriptome data with established protein interaction information, aiming to elucidate gene functional regulatory mechanisms. WGCNA is widely employed to construct gene co-expression networks, identify functionally related gene modules with similar expression patterns. In this study, we employed PPI network analysis to identify key regulatory genes. The results demonstrated that a total of 10 genes–*Tlr2, Tnf, Cxcl2, Cxcl10, Jun, Cxcl1, Ccl6, Il6, Il1b, and Ccl7* were identified as statistically significant candidates. Utilizing WGCNA, we selected the green, purple and yellow modules, which exhibited the highest correlations, and obtained 700 genes. Subsequently, we intersected the above acquired genes with pyroptosis-related DEGs, and finally obtained three key hub genes- *Tlr2, Tnf*, and *Il1b*. Zheng et al. (18) reported that diallyl disulfide could attenuate pyroptosis in HIBD neonatal rats via Il1b signaling pathway. Previous studies have also reported that tnf, a potent pro-inflammatory cytokine, is associated with the pathogenesis of various brain injury-related diseases (36–38). Wang et al. (39) investigated the therapeutic potential of certolizumab pegol (CZP), a monoclonal antibody targeting TNF-α, in a mouse model of middle cerebral artery occlusion (MCAO). The study demonstrated that early-stage administration of CZP significantly attenuated microglial activation and reduced the release of proinflammatory mediators, thereby effectively inhibiting microglial pyroptosis following ischemic stroke. Furthermore, regarding Tlr2, Zhang et al. (40) demonstrated a significant upregulation of Tlr2 in the hippocampal structure of neonatal rats following HIBD. In our study, the GEO dataset was based on unilateral brain tissue, revealing a marked increase in Tlr2 expression. These results indicate that Tlr2 plays a significant role in HIBD.

This study demonstrates significant remodeling of the immune microenvironment in HIBD. Our findings reveal distinct alterations in immune cell composition, aligning with prior studies that implicate immune dysregulation in neonatal HIBD pathogenesis (41). Compared to controls, the HIBD group exhibited pronounced inflammatory features involving both innate and adaptive immune responses. Specifically, we observed elevated levels of CD8+ naïve T cells, M0 macrophages, follicular CD4+ T cells, Th1 cells, and activated dendritic cells (DCs) in HIBD samples.

The enrichment of M0 macrophages suggests the initiation of nonspecific inflammatory responses during early injury, whereas the increase in Th1 cells-along with their secretion of pro-inflammatory mediators (e.g., IFN-γ and TNF-α)-likely exacerbates the neuroinflammatory milieu. Marina Seitz et al. (42) reported that the HIBD model induces an increase in peripheral macrophages, and hypothermia modulates myeloid cell polarization in HIBD. Furthermore, the infiltration of activated DCs underscores aberrant immune activation in HIBD, which may promote secondary neuronal injury via TLR receptor overexpression driven by damage-associated molecular patterns (DAMPs). Dendritic Cells (DCs) are the most powerful professional antigen-presenting cells (APCs) in the immune system. Previous studies have found that various central nervous system diseases are related to the activation of dendritic cells (43, 44). Notably, Li et al. (45) evaluated the association between ferroptosis-related genes and immune cells, similarly observing significant alterations in CD4+ T cells. However, notable discrepancies exist between their findings and our current results. These differences may be attributed to the distinct regulatory relationships between genes and immune cells across different programmed cell death pathways.

Additionally, the diagnostic utility of the identified hub genes associated with focal cell death in HIBD was validated using a random forest machine learning model. The model demonstrated that these genes possess significant discriminatory power, with variable importance measures (VIM) all surpassing the critical threshold of 1. Notably, the three genes exhibited high AUC values (> 0.75) in ROC analysis, further confirming their robust association with HIBD pathogenesis and reinforcing their reliability as potential molecular biomarkers. These findings suggest that the selected genes involved in PRDEGs may serve as key diagnostic indicators for HIBD, highlighting both their mechanistic significance and clinical translational value.

The biological significance of miRNA prediction mainly lies in revealing the fine regulatory mechanism of gene expression and its key role in neural development, and disease occurrence. Previous study has shown that TLR4 was proved to be the miR-326-3p directly target gene (46). Liping Chen (47) et al. reported that Aloesin ameliorates HIBD in neonatal mice by suppressing TLR4‐mediated neuroinflammation. A previous study has reported that the downstream target gene of miR-7031-5P is Wnt7a (48). In neonatal mice with hypoxic injury, oligodendrocyte-endothelial cell interactions regulate white matter vascular development in a Wnt-dependent manner. The loss of Wnt7a function may attenuate the angiogenic response to hypoxia, leading to severe white matter damage (49). The latest study indicates that Mapk10 is the target gene of miR-1894-3P (50). Treg cell-derived exosome miR-709 can alleviate pyroptosis of microglia after spinal cord injury (51). In addition, lncRNA Mtss1 promotes inflammatory response after intracerebral hemorrhage in mice by targeting miR-709 (52). Based on the current literature and our analytical findings, targeted miRNA modulation represents a promising therapeutic strategy for regulating pyroptosis in neonatal HIBD. Given the important role of *Tlr2, Tnf*, and *Il1b* in HIBD, we screened 136 potential drugs that target these three genes. By combining scoring methods and molecular docking techniques, muramyl dipeptide was selected. The current existing research on the role of MDP mainly focuses on inflammatory responses. Sanami Takada et al (53) reported that MDP is the ligand of nucleotide-binding oligomerization domain 2 (NOD2). In Blau Syndrome, the combination of the two leads to the upregulation of pro-inflammatory cytokines. However, Adham Fani Maleki (54) et al. reported that MDP treatment exerted various therapeutic effects in experimental autoimmune encephalomyelitis (EAE) mouse models of multiple sclerosis (MS). To date, the precise mechanistic role of muramyl dipeptide MDP HIBD pathogenesis remains unexplored. Through integrated bioinformatics analysis and molecular docking simulations, our preliminary findings suggest that MDP may participate in HIBD pathophysiology.

The major strength of this study lies in our innovative integration of bioinformatics and machine learning approaches, which systematically reveals for the first time the potential association between pyroptosis and HIBD. More importantly, through comprehensive analyses including miRNA target prediction and molecular docking, we have not only identified key regulatory genes but also provided verifiable molecular hypotheses and potential therapeutic targets for subsequent experimental investigations. his study has several limitations. First, although we conducted *in vivo* experiments to validate the association between pyroptosis-related genes and histopathological features of HIBD, further experimental evaluation is needed to elucidate the specific upstream/downstream regulatory factors of these hub genes and their spatial distribution across different brain regions. Second, the precise mechanisms underlying the interaction between pyroptosis-related genes and immune cell infiltration require more detailed investigation. Nevertheless, our findings provide a foundation for further exploration of the pathogenesis of HIBD.

## 5 Conclusion

In summary, our comprehensive transcriptomic analysis elucidated the transcriptional landscape underlying hypoxic-ischemic pathogenesis and systematically investigated potential molecular targets for therapeutic intervention. Through integrative bioinformatics analysis and rigorous diagnostic efficacy evaluation, we have delineated specific pathological mechanisms and identified promising molecular targets in HIBD, particularly those implicated in cytokine-cytokine receptor interactions and immune response pathways, which require further experimental validation. These findings provide valuable insights that advance our understanding of HIBD pathophysiology and contribute to the development of precision therapeutic approaches, thereby facilitating more targeted and effective clinical interventions for this debilitating condition.

## Data Availability

The datasets presented in this study can be found in online repositories. The names of the repository/repositories and accession number(s) can be found in the article/**Supplementary Material**.
