# Supplementary material for: Identifying pyroptosis-hub genes and immune infiltration in neonatal hypoxic-ischemic brain injury
Source: Front Immunol. 2025 Sep 5;16:1616312. doi: 10.3389/fimmu.2025.1616312 (PMC12446038; doi:10.3389/fimmu.2025.1616312)
Supplement: Supplementary file 1 [file Supplementaryfile1.docx]

Figure S1

Original data graph of TTC staining


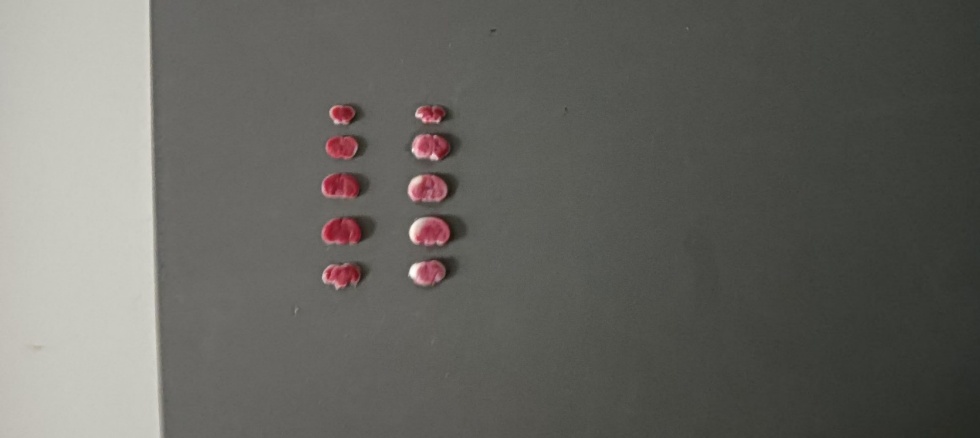


Figure S2

Original data graphs of HE staining


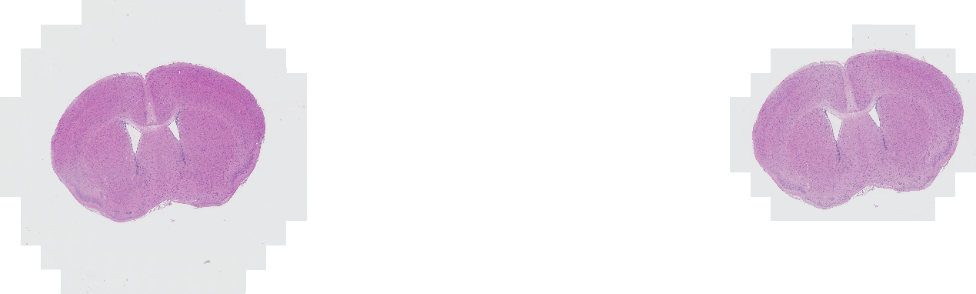


Sham


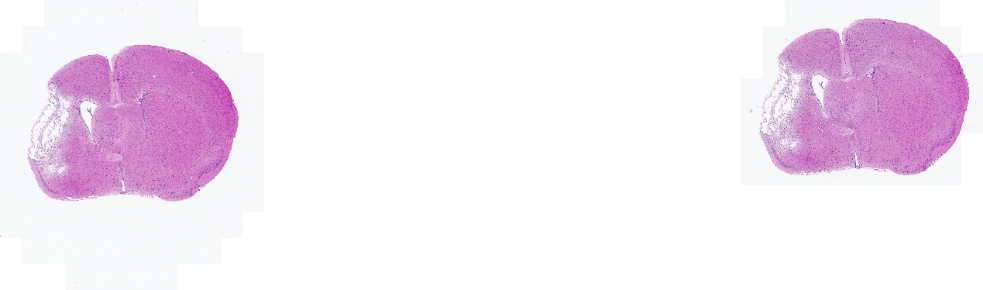


HIBD

Table S1

Original data of qRT-PCR

|  | Relative mRNA level of Tnf | Relative mRNA level of Il1b | Relative mRNA level of TLR2 |
| --- | --- | --- | --- |
| Sham1 | 0.01514 | 0.6018 | 1.9027 |
| Sham2 | 0.02472 | 0.4705 | 3.2982 |
| Sham3 | 0.02090 | 0.6393 | 3.7463 |
| Sham4 | 0.03816 | 0.7738 | 4.0909 |
| HIBD1 | 0.2432 | 2.6625 | 3.3629 |
| HIBD2 | 0.1547 | 2.5529 | 6.3325 |
| HIBD3 | 0.2466 | 2.7700 | 19.1786 |
| HIBD4 | 0.1769 | 3.0617 | 13.3035 |

Figure S3

Original data graphs of IF staining


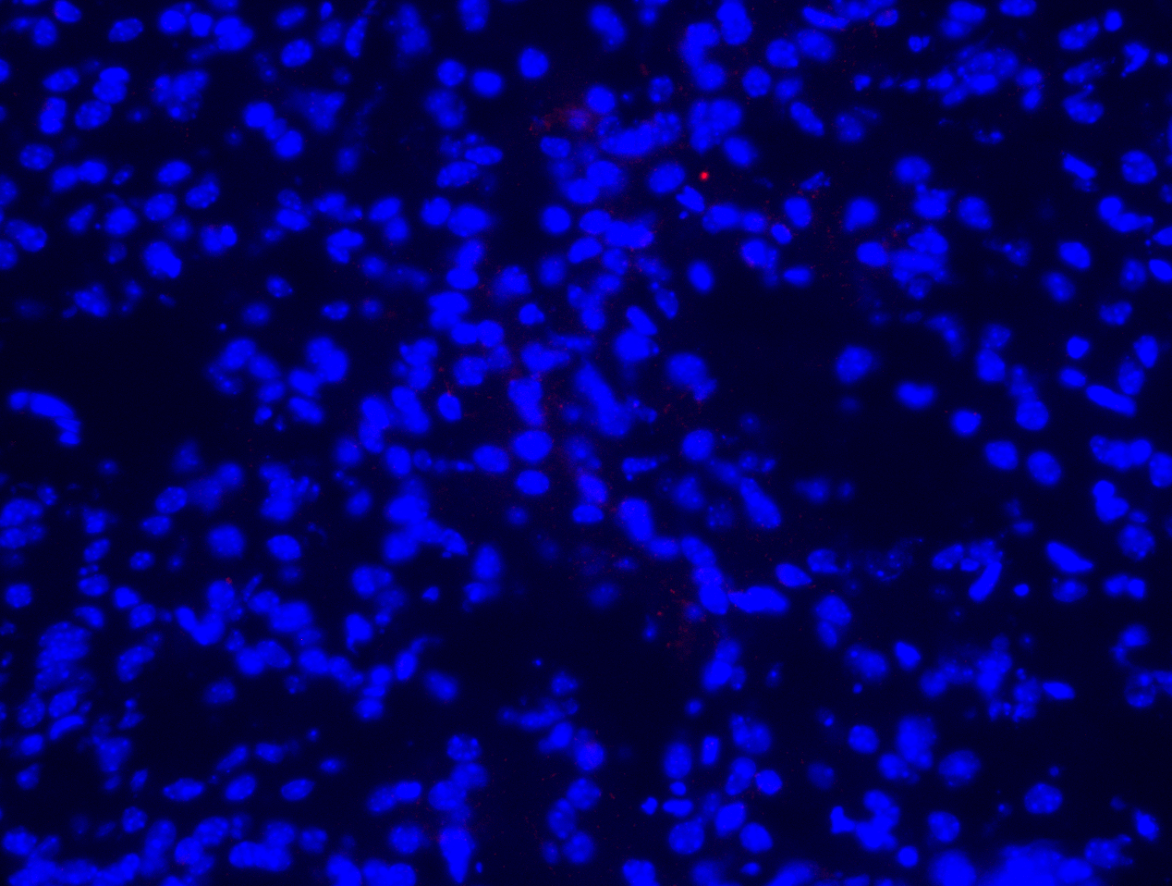


Tnf-Sham


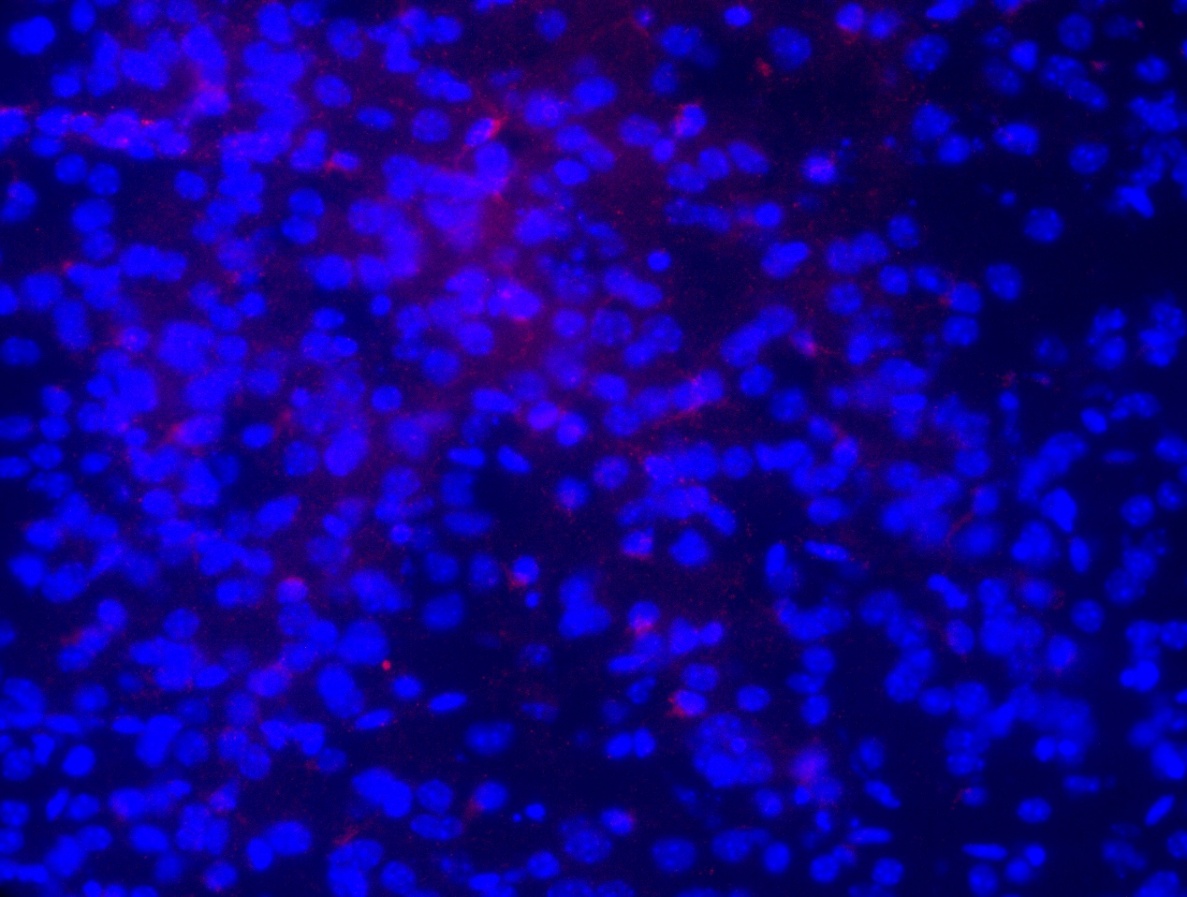


Tnf-HIBD


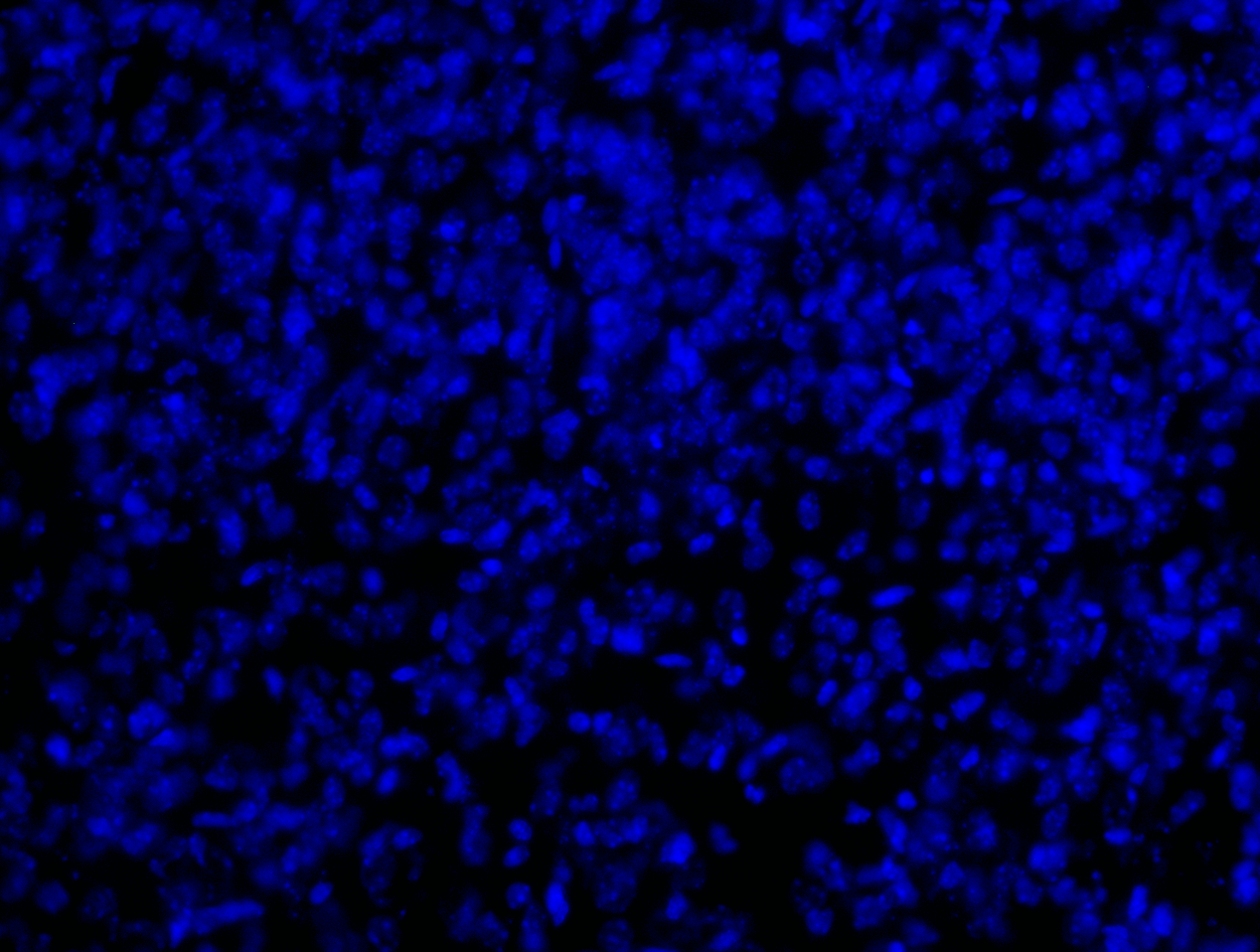


Il1b-Sham


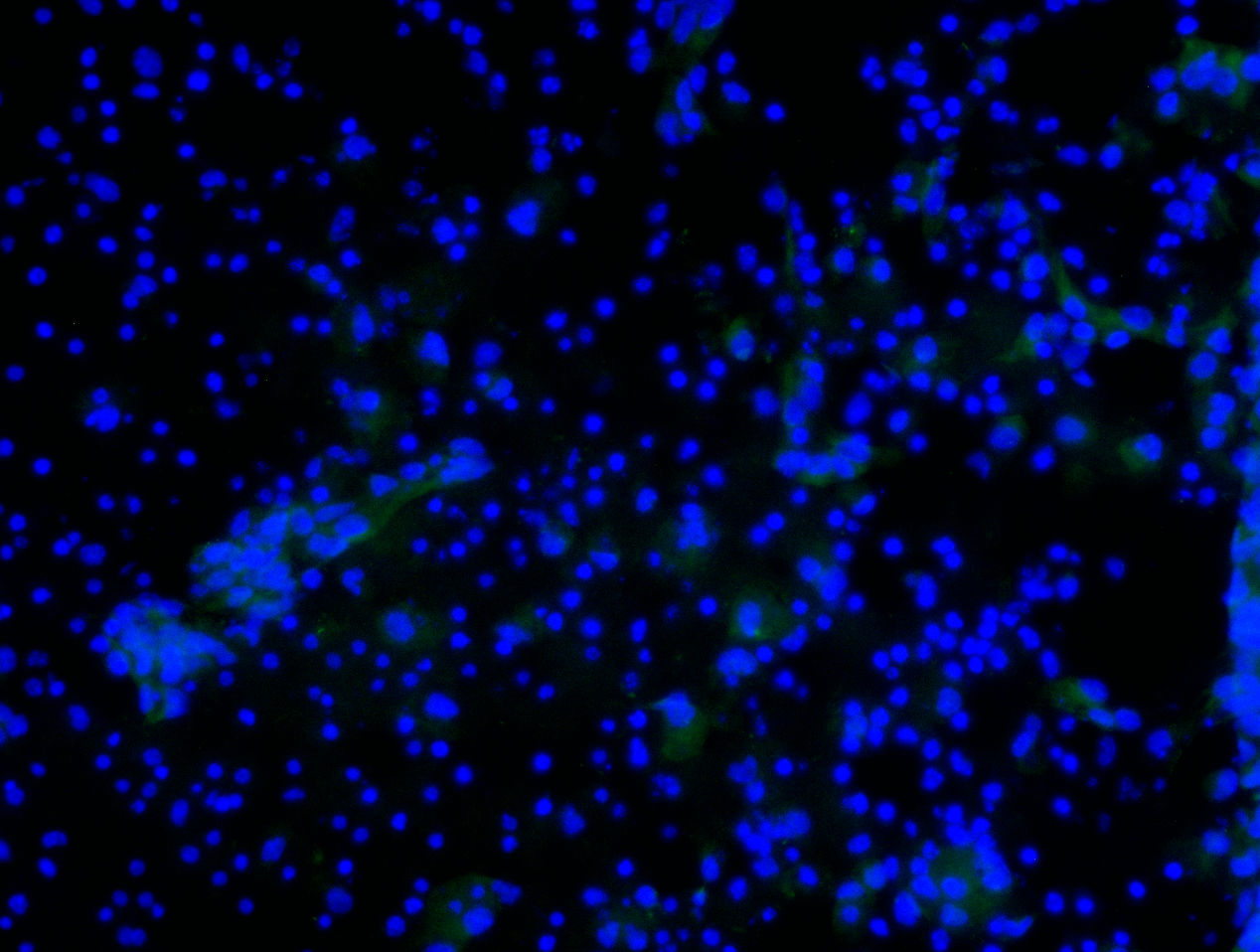


Il1b-HIBD


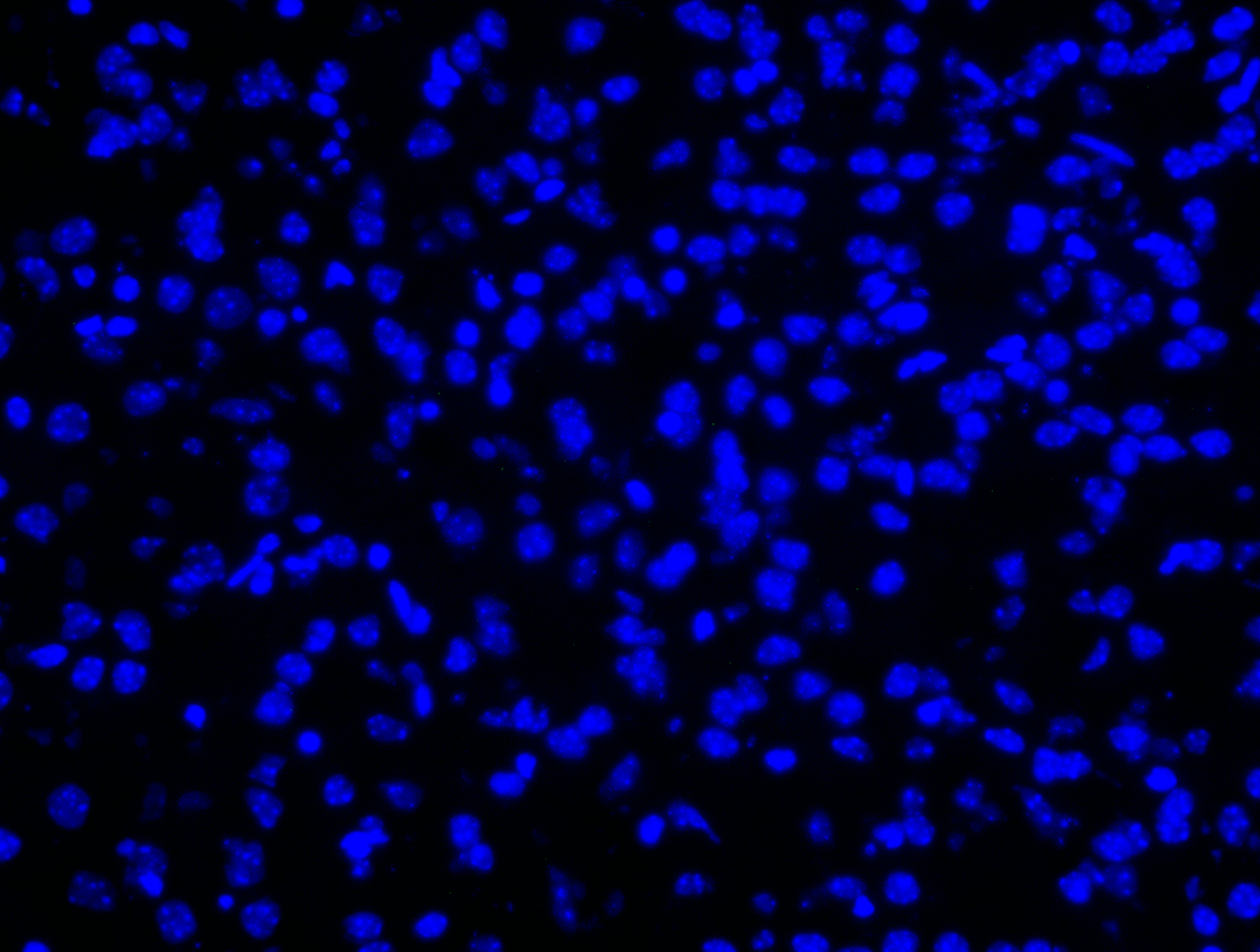


Tlr2-Sham


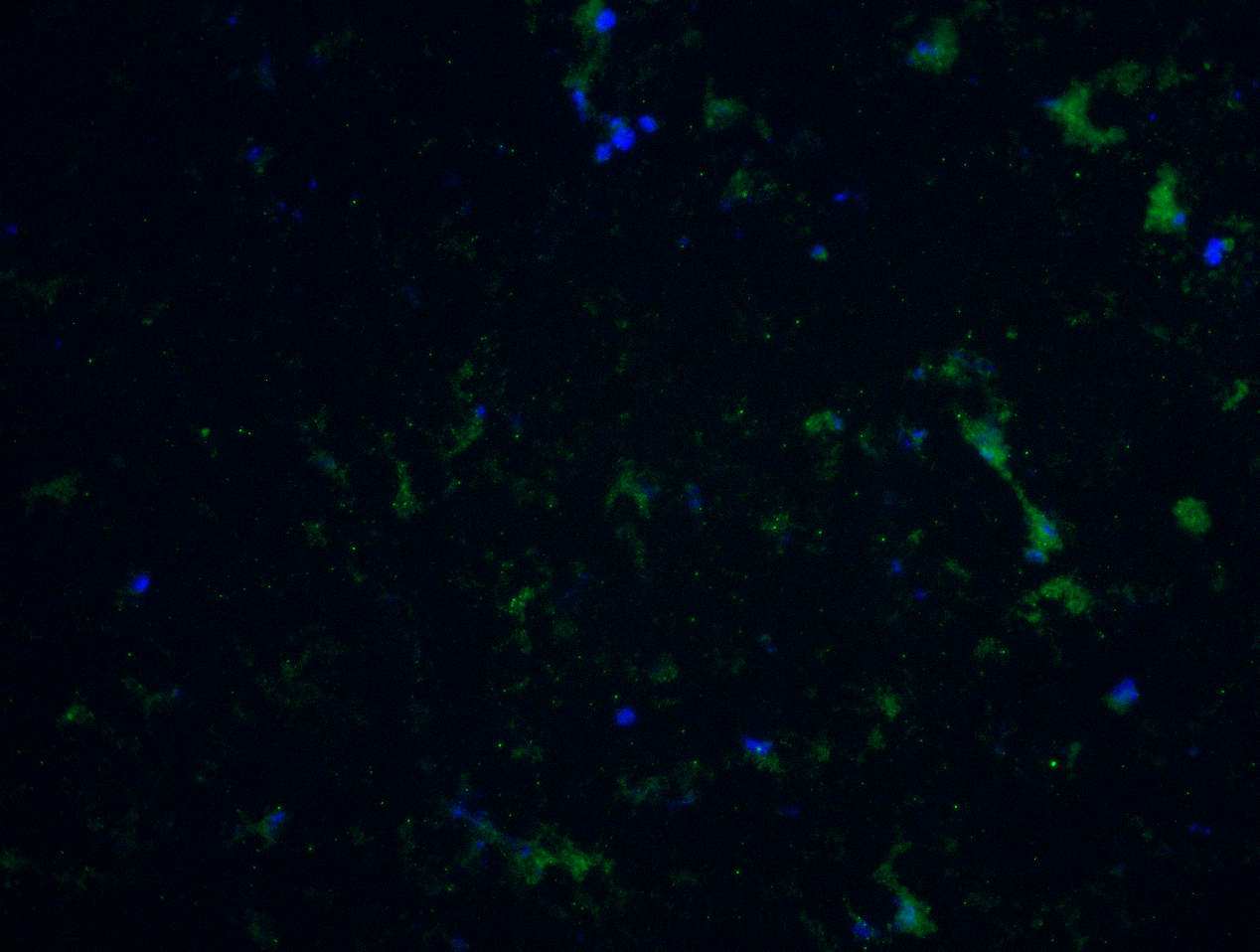


Tlr2-HIBD
