# Supplementary material for: Identifying pyroptosis-hub genes and immune infiltration in neonatal hypoxic-ischemic brain injury
Source: Front Immunol. 2025 Sep 5;16:1616312. doi: 10.3389/fimmu.2025.1616312 (PMC12446038; doi:10.3389/fimmu.2025.1616312)
Supplement: Supplementary file 2 [file Table1.docx]

**Supplementary materials**

**Table S1 The characteristics of the datasets.**

| **Dataset** | **Species** | **Type** | **No. of Samples (HIBD/Control)** | **Experimental model** |
| --- | --- | --- | --- | --- |
| GSE144456 | Mus musculus | mRNA | 24/8 | Unilateral carotid artery  ligation+hypoxia (8% oxygen,  40 min) |

**Table S2. The 176 pyroptosis-related genes.**

| No. | Gene name | No. | Gene name | No. | Gene name | No. | Gene name | No. | Gene name |
| --- | --- | --- | --- | --- | --- | --- | --- | --- | --- |
| 1 | TLR4 | 62 | CCR5 | 122 | GSDME | 183 | MIR125A | 244 | FAM3A |
| 2 | NLRP3 | 63 | IFNB1 | 123 | PLCG1 | 184 | NLRP12 | 245 | MIR106A |
| 3 | NFE2L2 | 64 | TNC | 124 | MIR223 | 185 | MIR1A-1 | 246 | HSPA12A |
| 4 | TNF | 65 | DNM1L | 125 | FTO | 186 | MIR96 | 247 | TRIM59 |
| 5 | CASP1 | 66 | CDC42 | 126 | ADAR | 187 | IRF2 | 248 | USP1 |
| 6 | CASP3 | 67 | XIST | 127 | NEAT1 | 188 | TUG1 | 249 | ARL2 |
| 7 | STAT3 | 68 | TRAF6 | 128 | SARM1 | 189 | MIR144 | 250 | YTHDC1 |
| 8 | TLR2 | 69 | TXNIP | 129 | MIR122 | 190 | ANGPTL2 | 251 | MIR326 |
| 9 | HIF1A | 70 | PRDM1 | 130 | CAMP | 191 | MIR199A-1 | 252 | TRIM29 |
| 10 | SIRT1 | 71 | XBP1 | 131 | KLF5 | 192 | APOC3 | 253 | GM12610 |
| 11 | HMGB1 | 72 | IRF1 | 132 | TIMP2 | 193 | MIR140 | 254 | EHF |
| 12 | IL1B | 73 | UCP2 | 133 | CYLD | 194 | GNA12 | 255 | FENDRR |
| 13 | PPARG | 74 | TET2 | 134 | TXN1 | 195 | STX4A | 256 | MIR486 |
| 14 | AKT1 | 75 | CTSB | 135 | CFLAR | 196 | ALKBH5 | 257 | CLMP |
| 15 | NFKB1 | 76 | C5AR1 | 136 | IL1 | 197 | HOTAIR | 258 | ALPK1 |
| 16 | PECAM1 | 77 | PIK3CG | 137 | TBXA2R | 198 | LILRB4A | 259 | ADGRA2 |
| 17 | MTOR | 78 | SIRT6 | 138 | PGK1 | 199 | PRDX3 | 260 | VPS4A |
| 18 | MMP9 | 79 | SPHK1 | 139 | IRAK1 | 200 | MIR20A | 261 | APBA3 |
| 19 | HMOX1 | 80 | METTL3 | 140 | HOMER1 | 201 | PVT1 | 262 | PHLPP2 |
| 20 | STING1 | 81 | SMARCA4 | 141 | PRMT5 | 202 | MIR200C | 263 | SMC4 |
| 21 | TLR9 | 82 | AIM2 | 142 | MIR145A | 203 | USF2 | 264 | TBXAS1 |
| 22 | CYBB | 83 | MAP3K7 | 143 | BHLHE40 | 204 | CDK9 | 265 | KIF23 |
| 23 | PPARGC1A | 84 | NTN1 | 144 | NEDD4 | 205 | SIRT4 | 266 | MIR101B |
| 24 | IL18 | 85 | SP1 | 145 | PSMB8 | 206 | MAP3K11 | 267 | MIR202 |
| 25 | P2RX7 | 86 | HSF1 | 146 | ATP6AP2 | 207 | PHLDA1 | 268 | LETMD1 |
| 26 | BAX | 87 | CD38 | 147 | SESN2 | 208 | PPP1CA | 269 | AASS |
| 27 | ADIPOQ | 88 | NT5E | 148 | HK2 | 209 | RND3 | 270 | CHMP4B |
| 28 | CASP4 | 89 | BECN1 | 149 | MIR214 | 210 | ACSS2 | 271 | DCLK2 |
| 29 | GSDMD | 90 | ALDH2 | 150 | ATF2 | 211 | DDX3X | 272 | MIR421 |
| 30 | FOXO3 | 91 | KEAP1 | 151 | SIRT5 | 212 | NSD2 | 273 | MINK1 |
| 31 | IL1R1 | 92 | S100A9 | 152 | MIR150 | 213 | MIRLET7B | 274 | PELP1 |
| 32 | PARP1 | 93 | HDAC6 | 153 | PF4 | 214 | NLRP1A | 275 | ZFAS1 |
| 33 | MIR155 | 94 | CLEC7A | 154 | CIRBP | 215 | ORMDL3 | 276 | TRIM16 |
| 34 | TREM2 | 95 | PKM | 155 | PPM1D | 216 | MIR18 | 277 | MIR590 |
| 35 | PIK3R1 | 96 | CGAS | 156 | MIR206 | 217 | NEK7 | 278 | GBP3 |
| 36 | TRPV4 | 97 | NLRC4 | 157 | SLC5A1 | 218 | GSDMA3 | 279 | PELI2 |
| 37 | CASP8 | 98 | EIF2AK2 | 158 | MIR17 | 219 | MARCHF1 | 280 | NINJ2 |
| 38 | DNMT1 | 99 | ROCK1 | 159 | PFKFB3 | 220 | TRIM27 | 281 | MIR199B |
| 39 | ATG5 | 100 | IKZF1 | 160 | IRAK3 | 221 | FOXQ1 | 282 | TXNRD3 |
| 40 | CEBPB | 101 | PCSK9 | 161 | METTL14 | 222 | TMEM59 | 283 | RCN1 |
| 41 | LGALS3 | 102 | TNFAIP3 | 162 | MIR125B-1 | 223 | MIR141 | 284 | KATNAL1 |
| 42 | EGR1 | 103 | BRD4 | 163 | PDCD4 | 224 | AZGP1 | 285 | CMPK2 |
| 43 | MMP2 | 104 | NR1D1 | 164 | WNK1 | 225 | WEE1 | 286 | CALM4 |
| 44 | EZH2 | 105 | S1PR2 | 165 | MIR182 | 226 | MIR29B-2 | 287 | SUGT1 |
| 45 | KL | 106 | GJB1 | 166 | MIR378A | 227 | TOMM20 | 288 | TIFA |
| 46 | SIRT3 | 107 | TRPM2 | 167 | CMKLR1 | 228 | AOAH | 289 | GSDMC |
| 47 | KLF4 | 108 | TFAM | 168 | NFE2L1 | 229 | TNFRSF21 | 290 | ADARB2 |
| 48 | LCN2 | 109 | AQP5 | 169 | PPP1CC | 230 | CPEB4 | 291 | TRIM45 |
| 49 | GHRL | 110 | KDM1A | 170 | MIR181A-2 | 231 | NSD1 | 292 | MIR672 |
| 50 | H19 | 111 | IKBKG | 171 | PEBP1 | 232 | MIR30C-1 | 293 | NEWENTRY |
| 51 | PYCARD | 112 | GPX4 | 172 | CASP7 | 233 | MIR124A-1HG | 294 | MIR302C |
| 52 | RIPK3 | 113 | FOXM1 | 173 | PTTG1 | 234 | ERBIN | 295 | MIR654 |
| 53 | SOX10 | 114 | MIR29A | 174 | GSTP1 | 235 | MIR20B | 296 | ZFP532 |
| 54 | RIPK1 | 115 | CX3CL1 | 175 | HVCN1 | 236 | FCNA | 297 | TRIM52 |
| 55 | CNR2 | 116 | HDAC4 | 176 | KMT2D | 237 | NAT10 | 298 | AU020206 |
| 56 | RORA | 117 | FNDC5 | 177 | GAS5 | 238 | MIR149 | 299 | MIR223HG |
| 57 | CEBPA | 118 | ANGPTL4 | 178 | ZBP1 | 239 | MARCHF5 | 300 | MIR1955 |
| 58 | DNMT3A | 119 | MALAT1 | 179 | KCNQ1OT1 | 240 | MIR199A-2 | 301 | GM41610 |
| 59 | PTPN11 | 120 | TREM1 | 180 | NLRP6 | 241 | CLEC5A |  |  |
| 60 | MDM2 | 120 | MEG3 | 181 | NPAS4 | 242 | MPEG1 |  |  |
| 61 | FGF10 | 121 | ABCG1 | 182 | TRIM72 | 243 | BRCC3 |  |  |
